# Multi‐Omics Analysis Reveals Impacts of LincRNA Deletion on Yeast Protein Synthesis

**DOI:** 10.1002/advs.202406873

**Published:** 2025-02-14

**Authors:** Ling Qin, Yuyang Pan, Songlyu Xue, Zhibo Yan, Chufan Xiao, Xiufang Liu, Dan Yuan, Jin Hou, Mingtao Huang

**Affiliations:** ^1^ School of Food Science and Engineering South China University of Technology Guangzhou 510641 China; ^2^ State Key Laboratory of Microbial Technology Shandong University Qingdao 266237 China

**Keywords:** lincRNAs, metabolic engineering, protein secretion, rapid growth, ribosomal proteins (RPs)

## Abstract

Non‐coding RNAs (ncRNAs) are widespread across various genomic regions and play a crucial role in modulating gene expression and cellular functions, thereby increasing biological complexity. However, the relationship between ncRNAs and the production of heterologous recombinant proteins (HRPs) remains elusive. Here, a yeast library is constructed by deleting long intergenic ncRNAs (lincRNAs), and 21 lincRNAs that affect α‐amylase secretion are identified. Targeted deletions of SUT067, SUT433, and CUT782 are found to be particularly effective. Transcriptomic and metabolomic analyses of the top three strains indicate improvements in energy metabolism and cytoplasmic translation, which enhances ATP supply and protein synthesis. Moreover, a yeast strain, derived from the SUT433 deletion, that can secrete ≈4.1 g L⁻^1^ of α‐amylase in fed‐batch cultivation through the modification of multiple targets, is engineered. This study highlights the significant potential of lincRNAs in modulating cellular metabolism, providing deep insights and strategies for the development of more efficient protein‐producing cell factories.

## Graphical abstract

This study demonstrates that the deletion of specific lincRNAs in yeast affects protein secretion. An engineered yeast strain, with multiple genetic modifications including lincRNA deletion, achieves a high yield of 4.1 g L⁻^1^ in fed‐batch cultivation. Multi‐omics analyses identify key lincRNAs that enhance energy metabolism and protein synthesis, providing a promising strategy for developing highly efficient protein‐producing yeast strains.

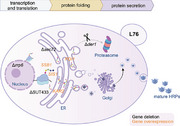

## 1 Introduction

The market for recombinant originator proteins reached $271 billion in 2021.^[^ ^1^ ^]^ The yeast *Saccharomyces cerevisiae* serves as one of the preferred platforms for producing various biologically active substances, including but not limited to heterologous recombinant proteins (HRPs), biopharmaceutical, biochemical, etc.^[^ ^2^, ^3^, ^4^ ^]^ However, enhancing HRP secretion in yeast is challenging due to the increased cellular stress it causes.^[^ ^5^, ^6^ ^]^ To mitigate this stress, various strategies have been employed: promoter engineering,^[^ ^7^ ^]^ signal peptide engineering,^[^ ^8^, ^9^, ^10^ ^]^ endoplasmic reticulum (ER) engineering,^[^ ^11^ ^]^ secretory pathway engineering,^[^ ^8^, ^12^, ^13^ ^]^ and cell wall engineering.^[^ ^14^ ^]^ Recent advancements, such as irrational mutagenesis,^[^ ^15^, ^16^ ^]^ and RNA interference,^[^ ^17^ ^]^ along with high‐throughput screening, have shown potential in constructing strains with enhanced protein secretion capabilities. The pcSecYeast model also offers predictive insights for improving HRP secretion.^[^ ^18^ ^]^ However, these methods primarily focus on gene‐level modifications,^[^ ^6^, ^19^ ^]^ which may burden cells with altered gene dosages. Therefore, non‐coding sequences, which constitute 25% of the yeast genome, warrant further attention, because they may offer additional regulatory options.^[^ ^20^ ^]^

In mammals, long non‐coding RNAs (ncRNAs) play diverse roles, including post‐transcriptional regulation, protein complex assembly, cell‐cell signaling facilitation, and modulation of protein allosteric regulation.^[^ ^21^ ^]^ Over the past decade, more than 2 000 transcription‐capable ncRNAs have been identified in yeast.^[^ ^20^, ^22^, ^23^ ^]^ A quantitative analysis of RNA expression on both strands of the *S.cerevisiae* genome under various conditions was conducted using a tiling array. This analysis identified a total of 7 272 transcripts, comprising 5 171 validated or uncharacterized open reading frame transcripts, 847 stable unannotated transcripts (SUTs), and 925 cryptic unstable transcripts (CUTs).^[^ ^24^ ^]^ Large‐scale functional profiling has been conducted on SUTs and CUTs in the ∆*rrp6* yeast strain.^[^ ^20^ ^]^ Manipulating long intergenic non‐coding RNAs (lincRNAs), such as certain SUTs and CUTs, can fine‐tune gene expression.^[^ ^20^ ^]^ The deletion of specific SUTs and CUTs notably impacts processes such as respiration (∆SUT125, ∆SUT126, etc.), steroid biosynthesis (∆CUT494), and others.^[^ ^25^ ^]^ For instance, the ∆SUT125 strain has shown increased secretion of endogenous invertase.^[^ ^26^ ^]^ Additionally, this strain exhibits compromised growth under stress conditions with 5% ethanol and 2% glycerol.^[^ ^25^ ^]^ While the precise mechanisms by which lincRNAs confer advantages in yeast are not fully understood, the potential of lincRNAs to develop robust yeast strains with enhanced energy production and balanced metabolism is promising and warrants further exploration.

In this study, we created a yeast lincRNA deletion library targeting lincRNAs located in the intergenic regions of genes, especially those located at least 200 bp from the start codon. We facilitated deletions using in situ replacement with the *KanMX4* fragment and screened for changes in α‐amylase secretion. Variations in α‐amylase secretion levels were validated through a CRISPR‐Cas9 markerless deletion approach. Notably, the top three strains, ∆SUT067, ∆SUT433, and ∆CUT782, that exhibited increased α‐amylase secretion also showed faster growth. Muti‐omic analyses (transcriptomic and metabolomic) revealed enhancements in energy metabolism and cytoplasmic translation, which, in turn, contributed to ATP supply and protein synthesis. To further enhance HRP secretion, we increased rRNA transcription and improved protein folding within the ER. A strain engineered with combinatorial modifications, including deletion of SUT433, achieved a secretion capacity of 4.1 gL^−1^ of α‐amylase in fed‐batch cultivation.

## 2 Results

### 2.1 LincRNAs Mediated Improved Heterologous Protein Secretion

We conducted an analysis of 208 lincRNA sequences, including 143 SUTs, 59 CUTs, and six other ncRNAs, located in the intergenic regions of genes and positioned at least 200 bp from the start codon.^[^ ^20^ ^]^ The distribution of these lincRNAs was relatively even across all chromosomes, with most sequences ranging between 300 and 700 bp in length (Figure S1a,b, Supporting Information). Notably, compared with CUTs, SUTs had a greater GC content and lower single‐base structural free energy in their RNA secondary structure (Figure S1c,d, Supporting Information), suggesting a potentially longer cellular half‐life of SUTs.

**Figure 1:**
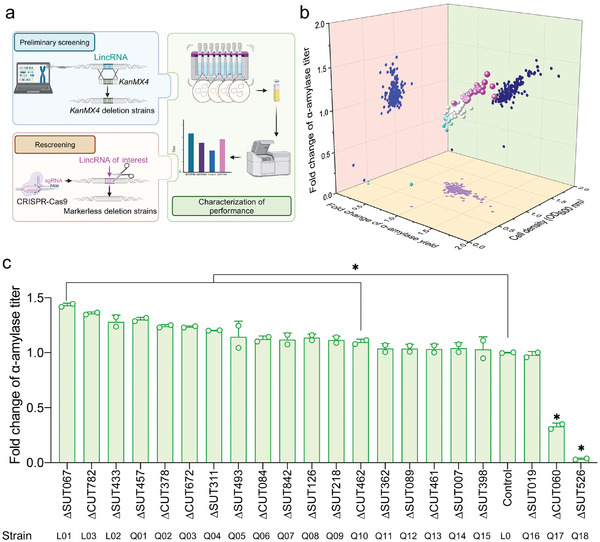
Screening lincRNAs in *S. cerevisiae* for enhanced α‐amylase secretion. a) Schematic workflow illustrating lincRNA deletions to increase α‐amylase secretion (created with BioRender.com). b) α‐Amylase secretion levels in strains with lincRNAs knockout using the *KanMX4* cassette, with AACK as the reference strain. c) α‐ Amylase secretion in strains with markerless lincRNA deletions using the CRISPR‐Cas9 system, with strain L0 as the control. Strains were cultivated in YTDP medium at 30°C for 96 h for α‐amylase production. Data shown are mean values ± SDs of biological duplicates or triplicates of single clones. The statistical significance was determined by a two‐tailed homoscedastic (equal variance) *t* test and indicated with an asterisk if *P* < 0.05.

To assess the influence of lincRNAs on the secretion capacity of HRPs, we used AACK as the reference strain, where α‐amylase is expressed through the plasmid CPOTud. This strain served as the basis for constructing a lincRNA *KanMX4* knockout library (**Figure** **1**a). The resulting library comprised 193 deletion strains. Screening these mutants revealed variable effects on α‐amylase secretion (Figure 1b). Specifically, over 10% of the deletion strains (21/193), which included 14 SUTs, 6 CUTs, and one other ncRNA, exhibited more than a 10% increase in α‐amylase secretion. The most effective deletion, ∆CUT782, led to an ≈40% increase in the α‐amylase titer. Conversely, the deletion of CUT060 and SUT526 resulted in substantial reductions in α‐amylase activity (by ≈70%) and biomass (by ≈60%), respectively. However, most lincRNA deletion strains did not significantly affect cell growth during preliminary screening (Figure 1b). These results indicate the importance of lincRNAs in modulating the secretion of HRPs.

To minimize potential interference from the *KanMX4* cassette insertion, which may affect the expression of transcriptionally adjacent genes,^[^ ^27^ ^]^ we reconstructed the lincRNA deletion strains using CRISPR‐Cas9 system. These newly engineered strains, based on targets identified in the preliminary screenings for altered amylase secretion, were further studied to confirm the initial findings. Among them, L01 (∆SUT067), L02 (∆SUT433), and L03 (∆CUT782) consistently showed more than a 20% increase in secretion (Figure 1c). Notably, the deletion of genes neighboring SUT067, SUT433, and CUT782 did not increase α‐amylase secretion (Figure S2a,b, Supporting Information). This confirms that the observed changes in α‐amylase secretion are directly attributed to the deletion of specific lincRNAs.

**Figure 2:**
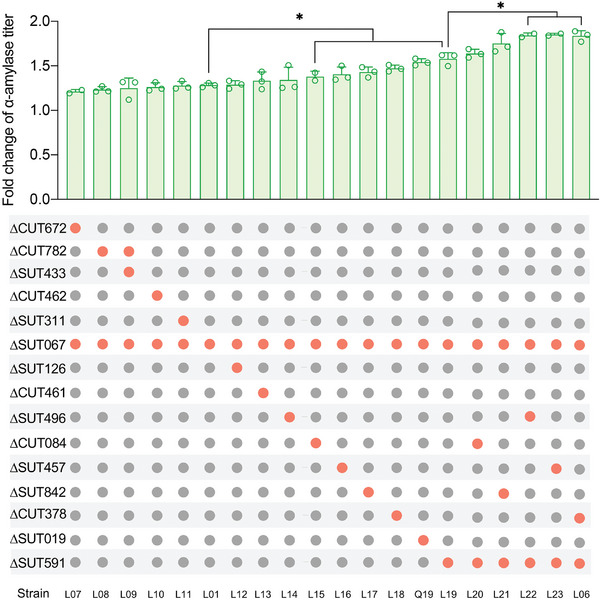
Combinatorial deletion of lincRNA and its impact on α‐amylase secretion. Strains were cultivated in SD‐2 × SCAA(+ura) medium at 30°C for 96 h to assess α‐amylase production. Strain L0 served as the control. Data shown are mean values ± SDs of biological duplicates or triplicates of single clones. The statistical significance was determined by a two‐tailed homoscedastic (equal variance) *t* test and indicated with an asterisk if *P* < 0.05.

Furthermore, to explore potential synergistic effects among the targeted lincRNAs in enhancing α‐amylase secretion, combinatorial deletions were generated. After two rounds of genetic manipulation, α‐amylase secretion in the L06 (∆SUT067∆SUT591∆CUT378), L22 (∆SUT067∆SUT591ΔSUT496), and L23 (∆SUT067∆SUT591ΔSUT457) strains increased by more than 80% (**Figure** **2**). However, the triple deletion strain L09 (∆SUT067∆SUT433∆CUT782) exhibited less enhancement of α‐amylase secretion compared with single deletions (Figure 2), suggesting that complex metabolic interactions are governed by lincRNAs.

### 2.2 Physiological Impacts of LincRNA Deletion in Strains

**Figure 3:**
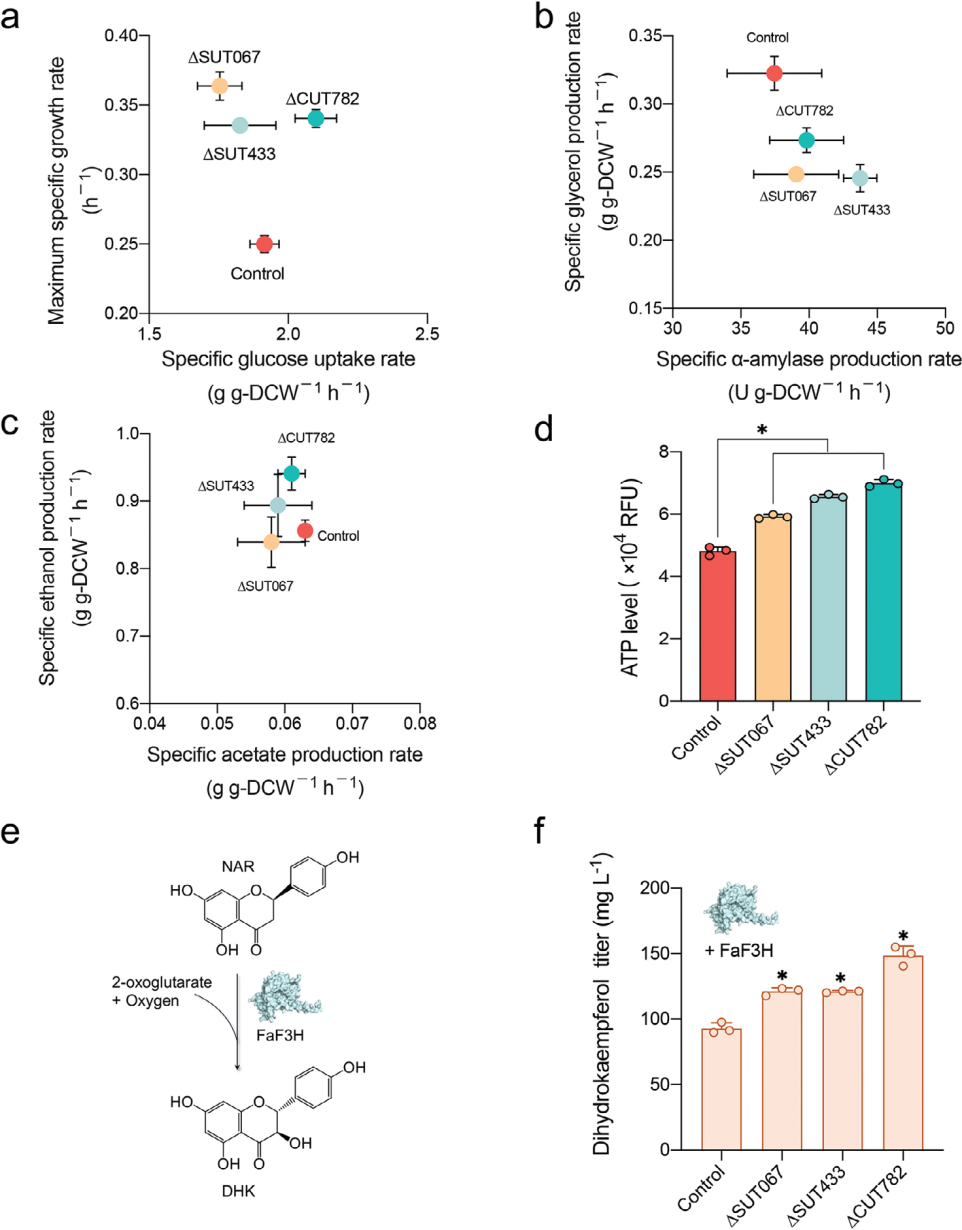
Physiological characteristics of the top three lincRNA‐deleted strains. a) Maximum specific growth rate on glucose and specific glucose uptake rate. b) Specific glycerol production rate and specific α‐amylase production rate. c) Specific ethanol production rate and specific acetate production rate. d) ATP content. e) Expression of *Fa*F3H (Q66MF0) based on the p416 plasmid. f) Dihydrokaempferol titers quantified after introducing the *Fa*F3H expression plasmid into control, ΔSUT067, ΔSUT433, and ΔCUT782 strains. Data shown are mean values ± SDs of biological triplicates. The statistical significance was determined by a two‐tailed homoscedastic (equal variance) *t* test and indicated with an asterisk if *P* < 0.05.

To better understand the impact of lincRNA deletions, we characterized additional physiological parameters of the top 3 strains ∆SUT067, ∆SUT433, and ∆CUT782. These strains had a greater maximum specific growth and achieved maximum cell density earlier than did the control (**Figure** **3**a; Figure S3a, Supporting Information). Additionally, the engineered strains exhibited an increased specific α‐amylase production rate and a decreased glycerol production rate (Figure 3b; Figure S3b, Supporting Information). A decrease in glycerol 3‐phosphate, the precursor for glycerol synthesis, was also observed in these engineered strains (Figure S3c, Supporting Information). Conversely, specific ethanol production rates were elevated in strains with ∆SUT433 and ∆CUT782 (Figure 3c; Figure S3d, Supporting Information), suggesting a redirection of carbon flow toward ethanol rather than glycerol during glycolysis in engineered strains. This redirection likely enhanced ATP synthesis, as confirmed by ATP assay results (Figure 3d). These results indicate that lincRNA deletions in yeast can lead to accelerated growth and increased ATP synthesis.

To assess their robustness, we overexpressed *Fragaria x ananassa* flavanone 3β‐hydroxylase (*Fa*F3H)^[^ ^28^ ^]^ in these strains using a plasmid (Figure S4, Supporting Information), which catalyzes the conversion of naringenin (NAR) to dihydrokaempferol (DHK) (Figure 3e).^[^ ^29^ ^]^ Notably, DHK production increased by 1.3‐fold in strains ∆SUT067 and ∆SUT433 and by 1.6‐fold in strain ∆CUT782 compared with the control (Figure 3f). These findings indicate that strains with lincRNA deletions exhibit enhanced robustness, making them promising candidates for industrial applications.

### 2.3 Multi‐Omics Analysis of the Rapid Growth Yeast Strains

We conducted a transcriptome analysis to identify differentially expressed genes (DEGs) between the top 3 strains (∆SUT067, ∆SUT433, and ∆CUT782) and the control strain during the exponential growth phase (Figure S5a, Supporting Information). Interestingly, the majority of DEGs in these strains were downregulated, potentially facilitating metabolic balance (Figure S5b, Supporting Information). We also observed a general decrease in the expression levels of genes involved in amino acid biosynthesis pathways in the top 3 strains (Figure S5c, Supporting Information). This suggests that the engineered strains might be conserving resources normally utilized for amino acid biosynthesis, possibly by assimilating amino acids directly from the medium. These findings are consistent with observations in other strains that exhibit enhanced protein synthesis^[^ ^30^ ^]^ This efficiency implies that the engineered strains may have adopted a more effective metabolic strategy to reduce their biosynthetic demands, particularly when environmental resources meet their needs. Kyoto Encyclopedia of Genes and Genomes (KEGG) enrichment analysis revealed that many genes in the glycolysis pathway were upregulated (Figure S6, Supporting Information).

**Figure 4:**
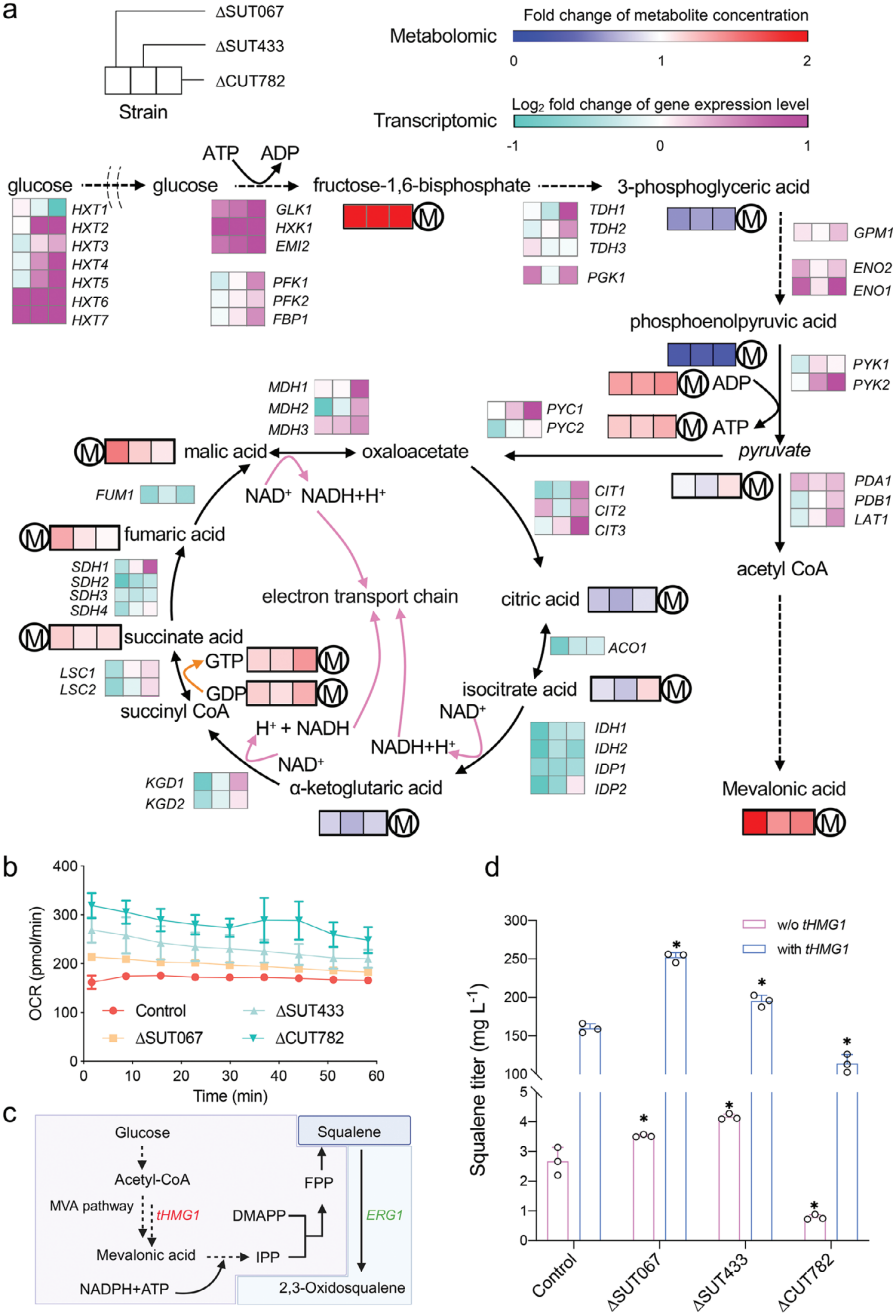
Multi‐omics analysis reveals fine‐tuning of energy metabolism. a) Transcriptional and metabolite changes in glycolysis and the TCA cycle in the top three strains L01(∆SUT067), L02(∆SUT433), and L03(∆CUT782) compared with the control L0; M represents relative metabolite changes. b) OCR was measured in cell densities of 5 × 10^5^ cells per well using a Seahorse FX‐96 oxygraph. C) The mevalonate pathway schematic shows the introduction of the *tHMG1* expression cassette for the squalene production, while Erg1 converts squalene into 2,3‐oxidosqualene. d) Squalene titers; data shown are mean values ± SDs of biological triplicates. The statistical significance was determined by a two‐tailed homoscedastic (equal variance) *t* test and indicated with an asterisk if *P* < 0.05.

We also examined gene expression and metabolic synthesis levels related to central carbon metabolism in the top three strains (**Figure** **4**a; Figure S7a, Supporting Information). The expression levels of genes associated with fructose‐1,6‐bisphosphate synthesis were significantly elevated, and the concentration of fructose‐1,6‐bisphosphate itself also increased, thereby ensuring enhanced carbon flux through the glycolysis pathway. There was a decrease in the transcription levels of genes associated with the tricarboxylic acid (TCA) cycle (Figure 4a). However, the metabolomics results showed that not only ATP and NADH, but also GDP and GTP levels were higher in the top three strains than in the control (Figure 4a; Figure S7b, Supporting Information). The oxygen consumption rate (OCR) of the top three strains was also enhanced (Figure 4b), reflecting improved oxidative phosphorylation in mitochondria. The increase in reactive oxygen species (ROS) in these strains could be attributed to the enhanced activity of the electron transport chain (Figure S7c, Supporting Information). These results clearly demonstrated that the enhancement of the glycolysis pathway and the generation of additional ATP contributed to the rapid growth of top three strains.

Furthermore, the enhancement of the glycolysis pathway facilitated an increased supply of mevalonic acid (Figure 4c), a key precursor for squalene synthesis. Indeed, deletion of SUT067 and SUT433 increased squalene production (Figure 4d), but the expression level of *ERG1* did not increase with ∆SUT067 (Figure S8, Supporting Information). Furthermore, upon overexpression of *tHMG1* (truncated *HMG1*),^[^ ^31^ ^]^ the increase in squalene production in ∆SUT067 and ∆SUT433 was significantly greater than that in the control (Figure 4d).

### 2.4 Enhancing the Translation Process Improved Protein Synthesis

**Figure 5:**
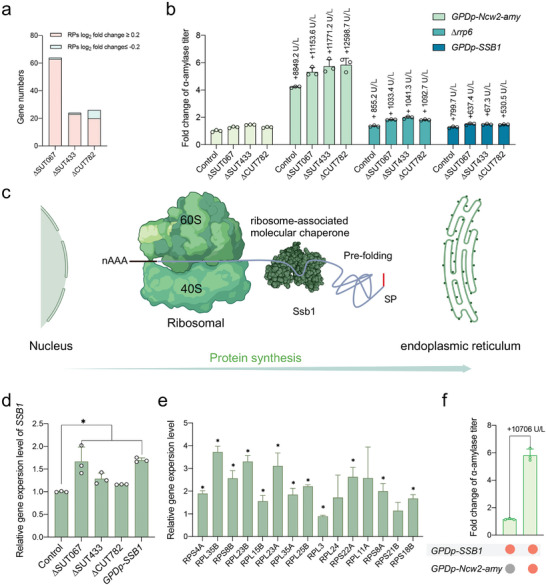
Enhanced expression of ribosome proteins supports protein synthesis. a) DEGs of ribosome proteins (significantly DEGs, *P*‐adj < 0.05 and |log_2_ fold change| ≥ 0.2) in the top three strains compared with the control strain. b) Changes in α‐amylase titer following the introduction of an additional amylase expression cassette, deletion of *RRP6*, or overexpression of *SSB1* in the top 3 strains (L01, L02, L03) and the control strain (L0). c) Schematic overview of the ribosome‐associated molecular chaperone Ssb1 (created with BioRender.com). d) Expression levels of *SSB1* gene in the strains, data are presented as mean ± SDs (n = 3). e) Relative gene expression levels of RPs in the *SSB1* overexpression strain (L62) compared with the control (L0). f) Introduction of an additional amylase expression cassette in the *SSB1* overexpression strain resulted in a higher increase in α‐amylase titer, with the starting strain L0 serving as the control. Strains were cultivated in SD‐2 × SCAA(+ura) medium at 30°C for 96 h, data shown are mean values ± SDs of biological triplicates. The statistical significance was determined by a two‐tailed homoscedastic (equal variance) *t* test and indicated with an asterisk if *P* < 0.05.

We further explored the reporter Gene Ontology (GO) terms for the top three strains. The top five reporter GO terms for each strain highlighted the key regulatory networks associated with rapid growth and increased protein secretion.^[^ ^30^ ^]^ Specifically, genes associated with the GO term “cytoplasmic translation” were upregulated in strains ∆SUT067 and ∆SUT433 (Figure S9, Supporting Information). The numbers of genes associated with transcriptional changes in ribosomal proteins (RPs) are shown (**Figure** **5**a). Among the strains, L01(∆SUT067) had a greater average transcriptional level for RPs than did L02(∆SUT433) and L03(∆CUT782) (Figure S10, Supporting Information). In rapidly growing yeast cells, ≈60% of total transcription is dedicated to ribosomal RNA, which accounts for ≈50% of RNA polymerase II transcription.^[^ ^32^ ^]^ This substantial transcriptional effort consumes significant cellular resources. We hypothesize that robust expression of RPs enhances protein synthesis, particularly when the transcription of the target protein is also increased. To test this hypothesis, we introduced an additional copy of the amylase gene with the NCW2 signal peptide into the XI‐3 site on the chromosome.^[^ ^9^ ^]^ The results indicated an increase in α‐amylase secretion across all strains, with notably greater increases observed in the top three strains than in the control (Figure 5b). These findings suggest that the enhanced translation capability of the top three strains is a key driver of their increased protein synthesis.

### 2.5 Engineering rDNA Transcription for Synergistic Adaptation in RP‐Increase Chassis

In contrast to the enhancements observed in cytoplasmic translation processes, several key processes, including rRNA processing and the biogenesis of ribosomal small and large subunits, were downregulated in the top three strains (Figure S9, Supporting Information). This suggests differential regulation of ribosomal assembly components, which may impact overall protein synthesis efficiency.

To assess the impact of ribosome biogenesis on α‐amylase secretion, we knocked out *DOT6* and *TOD6*, both of which encode proteins that act as transcriptional repressors of ribosome biogenesis genes (Figure S11a, Supporting Information).^[^ ^33^ ^]^ Their deletion slightly reduced α‐amylase secretion, but had no effect on the strain ∆SUT067 (Figure S11b, Supporting Information). Furthermore, overexpression of the transcriptional activator Sfp1, which directly activates genes involved in ribosome biogenesis and protein synthesis,^[^ ^33^ ^]^ did not influence α‐amylase secretion (Figure S11c, Supporting Information). Additionally, overexpressing Sch9, an AGC family protein kinase,^[^ ^34^ ^]^ in the ∆*tod6* strain resulted in decreased α‐amylase activity and cell density (Figure S11d,e, Supporting Information). These results suggest that attempts to enhance ribosome biogenesis through the TORC and cAMP pathways in the ∆SUT067 strain did not yield the anticipated outcomes.

To further enhance rDNA transcription, we deleted the 3′‐5′ nuclear exonuclease Rrp6, a component of nuclear exosomes involved in RNA processing and maturation.^[^ ^35^ ^]^ This deletion in the top three and control strains resulted in greater increases in α‐amylase secretion in the engineered strains than in the control strain (Figure 5b). Additionally, qRT‐PCR analysis revealed increased transcription levels of *SRD1*, *HMT1*, *RRN7*, and *RRN6* following the deletion of *RRP6* (Figure S12a, Supporting Information). Moreover, individual overexpression of genes related to rDNA transcription and modification,^[^ ^36^ ^]^ specifically *RRN7* and *HMT1*, led to increased α‐amylase secretion (Figure S12b, Supporting Information). These findings indicate that optimizing rDNA transcription can synergize with increased RPs to enhance protein secretion.

### 2.6 Enhancing RP Expression through Ssb1 Regulation in the Top Three Strains

The top three strains exhibited an enhanced translation capacity, which contributed to their rapid growth. Given that many cytoplasmic and cell membrane proteins bypass process in the ER, we focused on the prefolding process of nascent polypeptides within the cytoplasm. The ribosome‐associated molecular chaperone Ssb1 aids in the passage of the nascent polypeptide chain through the ribosome (Figure 5c).^[^ ^37^ ^]^ Overexpression of the *SSB1* gene resulted in enhanced α‐amylase secretion, although the increase in the engineered strains was lower than that in the control (Figure 5b). This diminished increase may be attributed to the robust pre‐folding capabilities already present in the top three strains. To further investigate this phenomenon, we conducted qRT‐PCR, which indicated an increased expression level of *SSB1* in both the top three and *SSB1* overexpression strains (Figure 5d). Interestingly, RP genes were also upregulated in the *SSB1*‐overexpressing strain (Figure 5e). Overexpressing *SSB1* led to increased α‐amylase production (Figure 5b). Similarly, introducing an additional copy of the amylase gene with the NCW2 signal peptide into the XI‐3 site in the *SSB1* overexpression strain resulted in greater increases in α‐amylase than in the control (Figure 5f). These findings suggest that *SSB1* upregulation in the top three strains is a key factor in enhancing the transcriptional levels of RP genes, contributing to increased protein synthesis. Therefore, Ssb1 could be a promising target for genetic engineering to increase protein secretion.

### 2.7 Combining LincRNA Targets to Design Efficient Protein Secretion Chassis Cells

**Figure 6:**
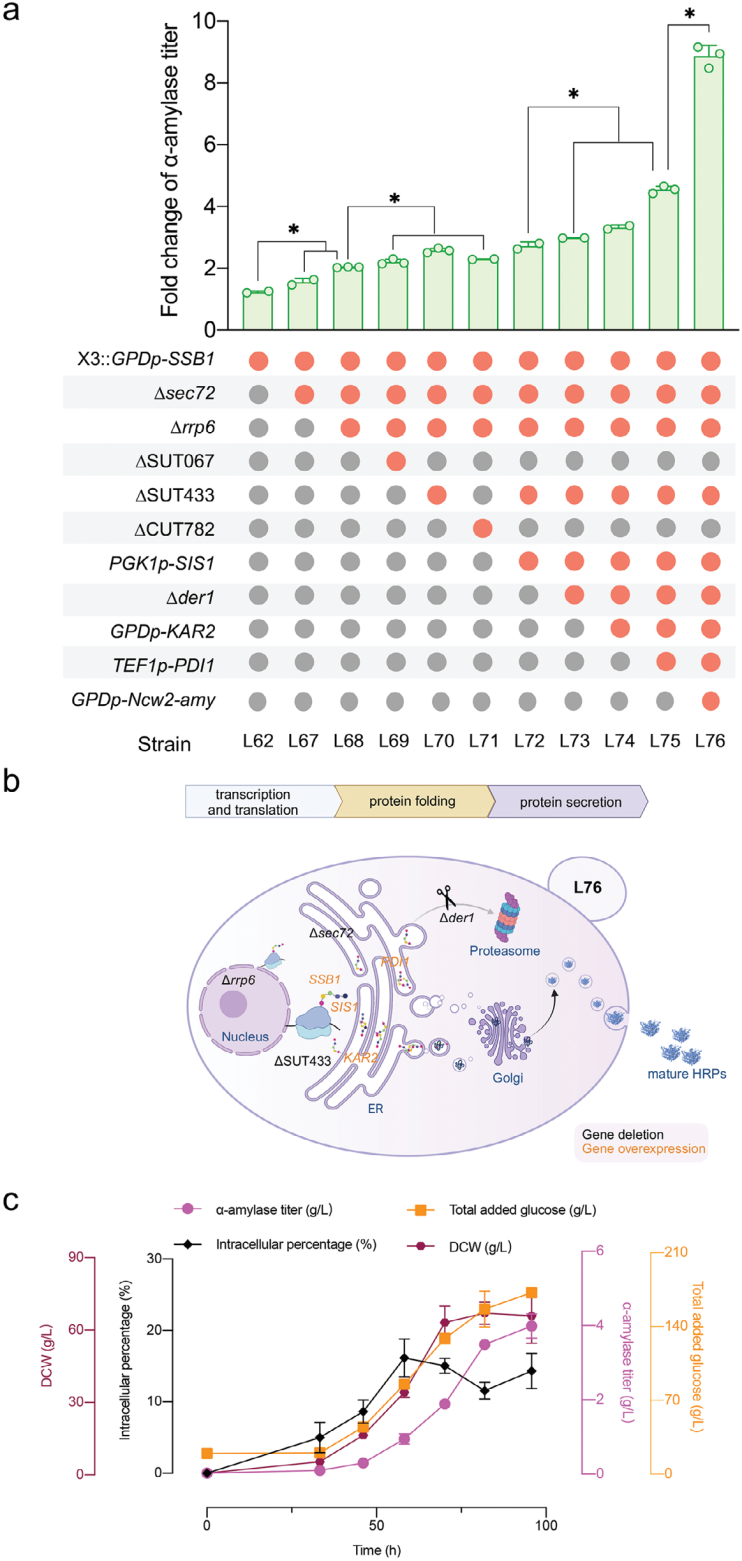
Enhanced transcription and translation promote protein secretion. a) α‐Amylase titers in engineered strains, with the starting strain L0 as the control. Strains were cultivated in SD‐2 × SCAA(+ura) medium at 30°C for 96 h for α‐amylase production, data shown are mean values ± SDs of biological duplicates or triplicates of single clones. b) Schematic workflow for genetic manipulation of *S. cerevisiae* to increase α‐amylase secretion (created with BioRender.com). c) Fed‐batch cultivation of strain L76. The statistical significance was determined by a two‐tailed homoscedastic (equal variance) *t* test and indicated with an asterisk if *P* < 0.05.

To further verify whether increased Ssb1 activity aids in protein secretion, we deleted Sec72, a protein with a TPR domain that binds to the Ssb1 ATPase domain and inhibits nucleotide exchange.^[^ ^38^ ^]^ The deletion of Sec72 led to a 23% increase in α‐amylase production compared with that of the L62 strain (**Figure** **6**a). Additionally, α‐amylase secretion in strain L68 increased by 33% after *RRP6* was deleted. A further increase in α‐amylase production was observed following the additional deletions of SUT067, SUT433, and CUT782 in the L68 strain (Figure 6a). Integrating the *SIS1* gene into strain L70 further increased α‐amylase production (Figure 6a). These combined genetic manipulations of lincRNA targets and conventional gene targets significantly enhanced α‐amylase production, expanding the range of potential protein secretion targets.

We hypothesize that the L72 strain likely has an enhanced capacity for cytoplasmic nascent peptide pre‐folding. The deletion of *SEC72* may assist in the translocation of nascent peptides into the ER.^[^ ^9^ ^]^ Deleting the *DER1* gene, which facilitates the transport of misfolded proteins through the ER membrane, led to an increase in α‐amylase production in comparison to that in strain L72 (Figure 6a). Furthermore, replacing the native promoter of Kar2, a chaperone protein,^[^ ^39^ ^]^ with the promoter *GPDp* resulted in a 13% increase in α‐amylase production compared with that of L73 (Figure 6a). Remarkably, overexpressing the protein disulfide isomerase Pdi1 by replacing its native promoter with the promoter *TEF1p* led to a 36% increase in α‐amylase production (Figure 6a). Compared with L0, strain L75 exhibited a 4.55‐fold increase in α‐amylase production.

Additionally, introducing an extra copy of the α‐amylase gene into the chromosome of L75 nearly doubled the secretion level in the resulting strain L76, significantly surpassing that of the control (Figure 6a,b; Figure S13a, Supporting Information). To test the versatility of the L75 strain in promoting HRP secretion, we replaced the α‐amylase expression plasmid with a human serum albumin (HSA) expression plasmid (Figure S13b, Supporting Information).^[^ ^30^ ^]^ This treatment led to a significant increase in HSA production in L78 compared with that in the control strain L80 (Figure S13c, Supporting Information). Our results suggest that a cell factory engineered with a lincRNA target holds promise for enhancing protein secretion.

### 2.8 Fed‐Batch Fermentation of Strain L76

To optimize α‐amylase production, strain L76 underwent fed‐batch cultivation in a bioreactor with controlled glucose feeding. The dissolved oxygen level was maintained above 25% to control medium feeding and prevent overflow metabolism. After 96 h, the α‐amylase titer reached 4.1 g L⁻^1^, demonstrating robust protein production (Figure 6c). The final cell density reached 66.1 g dry cell weight L^−1^ (g‐DCW L⁻^1^), with a total glucose consumption of 177.07 g L⁻^1^. (The dissolved oxygen levels are shown in S14, Supporting Information). These results highlighted the effectiveness of our engineered strains in HRP production.

## 3 Discussion

*S. cerevisiae*, often employed as a chassis cell, has been extensively engineered to produce value‐added products, including HRPs.^[^ ^2^ ^]^ Although gene‐level manipulations are becoming more common, their effects on production can sometimes be limited. To address this challenge, we screened a lincRNA deletion library using α‐amylase as an indicator of protein secretion. Our analysis of the GC content and secondary structure free energy of lincRNA sequences suggested that CUTs may have a shorter half‐life than SUTs (Figure S1c,d, Supporting Information). Large‐scale CRISPR library screenings revealed that disrupting the expression of lincRNAs can improve α‐amylase secretion.^[^ ^40^ ^]^ E.g., the ∆SUT126 strain promoted both endogenous protein expression and HRP secretion.^[^ ^26^ ^]^ Using CRISPR‐Cas9‐mediated deletion, we found that both CUT060 and SUT526 are crucial for yeast growth, suggesting that lincRNAs are involved in metabolic regulation. The combination of ΔSUT067 and ΔSUT591, when further combined with ΔSUT496, ΔSUT457, or ΔCUT378, led to a gradual increase in the secretion of α‐amylase. These results highlight the unique metabolic pathways influenced by each additional lincRNA deletion.

Multi‐omics analysis combined with specific determination of metabolite production rates revealed a reprograming of carbon flux through glycolysis, leading to enhanced respiration and TCA cycle activity in the top three strains. Redirection of metabolism can yield benefits. For instance, enhancing pentose phosphate pathway flux can increase the production of NADPH. Increased NADPH availability is crucial for supporting the synthesis of chemicals and fuels.^[^ ^41^ ^]^ The appeal of biomanufacturing lies in its ability to produce complex natural structures.^[^ ^2^ ^]^ For instance, the complete biosynthesis of QS‐21 in engineered yeast involves the expression of 38 heterologous enzymes.^[^ ^42^ ^]^ Our study shows that the top three strains facilitated the expression of exogenous enzymes from various sources. Adjusting the Mevalonate pathway fluxes and increasing ATP levels could broaden cell factory applications in natural product synthesis.

Ribosome availability is crucial for yeast cells to achieve fast growth rates, offering a competitive advantage.^[^ ^43^ ^]^ Our findings show that transcription factors such as Ssb1 upregulated RP gene expression in top three strains, significantly enhancing α‐amylase secretion. Moreover, the specific activity of β‐glucosidase can be increased by overexpressing the RPs Rpl8, Rpl13b, and Rpl16A.^[^ ^14^ ^]^ This finding suggests that targeting yeast translation processes could be a strategy for enhancing protein synthesis. Focusing on the Ssb1 target, with the deletion of *RRP6* and *SEC72*, we hypothesize that the engineered strain shows enhanced transcription and translation capabilities for HRP. Subsequent knockouts of SUT067, SUT433, and CUT782 led to further improvement in α‐amylase secretion capacity, likely due to increased cellular energy supply (Figure 6a). In fed‐batch cultivation, strain L76 achieved an α‐amylase production level of 4.1 g L⁻^1^, which is ≈65% higher than the previously reported titer of 2.5 g L⁻^1^.^[^ ^12^ ^]^ Additionally, the secretion capacity of HSA from the L78 strain increased fivefold, demonstrating the effectiveness of our approach in enhancing the production of this industrial strain.

In summary, our study highlights the potential of lincRNAs to fine‐tune metabolic pathway fluxes and facilitate protein synthesis, significantly enhancing the versatility of yeast as a cell factory. Considering that *S. cerevisiae* is a model organism, the targets and pathways we identified and engineered hold promise as foundational principles for designing and engineering other cell factories for efficient protein synthesis.

## 4 Experimental Section

### Strains and Culture Conditions

All strains used in this study are listed in Table S1 (Supporting Information). The strain *S. cerevisiae* CEN.PK 530–1CK (*MATa tpi1(41‐707)::loxP*) was used as the starting strain for deleting lincRNAs with the *KanMX4* fragment. The strain CEN.PK 530–1D (*MATa ura3‐52 tpi1(41‐707)::loxP‐KanMX4‐loxP*) was used as a starting strain for markerless deletion of lincRNAs based on the GTR‐CRISPR system.^[^ ^44^ ^]^

LB medium (5 g L^−1^ yeast extract, 10 g L^−1^ tryptone, and 10 g L^−1^ NaCl) was used to culture *Escherichia coli* DH5α with or without 100 µg mL^−1^ of ampicillin at 37 °C and 250 rpm. Synthetic complete without uracil (SD‐URA) medium (20 g L^−1^ glucose, 1.7 g L^−1^ yeast nitrogen base minus amino acids, 5 g L^−1^ (NH_4_)_2_SO_4_, 0.77 g L^−1^ complete supplement mixture (CSM, without uracil) was used for yeast strain selection and cultivation when necessary. YPD (10 g L^−1^ yeast extract, 20 g L^−1^ peptone, and 20 g L^−1^ glucose) medium was used for yeast strain cultivation with or without 200 µg mL⁻^1^ G418. YTD medium contains 10 g L^−1^ yeast extract, 20 g L^−1^ tryptone, 20 g L^−1^ glucose. YTDP (10 g L^−1^ yeast extract, 20 g L^−1^ tryptone, 20 g L^−1^ glucose, 13.6 g L^−1^ Na_2_HPO_4_·12H_2_O, and 9.7 g L^−1^ NaH_2_PO_4_·2H_2_O) and SD‐2 × SCAA^[^ ^15^ ^]^ (20 g L^−1^ glucose, 6.9 g L^−1^ yeast nitrogen base without amino acids, 1 g L^−1^ BSA, 13.6 g L^−1^ Na_2_HPO_4_·12H_2_O, and 9.7 g L^−1^ NaH_2_PO_4_·2H_2_O, 0.19 g L^−1^ Arg, 0.4 g L^−1^ Asp, 0.126 g L^−1^ Glu, 0.13 g L^−1^ Gly, 0.14 g L^−1^ His, 0.29 g L^−1^ Ile, 0.4 g L^−1^ Leu, 0.44 g L^−1^ Lys, 0.108 g L^−1^ Met, 0.2 g L^−1^ Phe, 0.22 g L^−1^ Thr, 0.04 g L^−1^ Trp, 0.052 g L^−1^ Tyr, and 0.38 g L^−1^ Val, pH = 6.0) were used for protein production. SD‐FOA medium (adds 1 g L^−1^ 5‐FOA and 50 mg L^−1^ uracil to SD‐URA medium) was used for URA3 marker elimination. A final concentration of 2% agar was used for solid medium.

### Plasmids, Primers, and Genetic Manipulations

The plasmids and primers used in this study are listed in Tables S2 and S3 (Supporting Information), respectively. For overexpression, plasmids p416‐GPD or p426‐GPD were digested with *Eco*RI and *Hin*dIII. Gene fragments were amplified by PCR with specific primers. These fragments were then ligated to the backbone using the Gibson cloning method.^[^ ^45^ ^]^ Codon‐optimized gene fragments of *Fa*F3H were synthesized by GenScript (Nanjing, China). The full expression cassette was assembled through fusion PCR and integrated with the plasmid p416 backbone, which was cleaved by *Sac*I and *Bam*HI. The plasmid maps are shown in Figure S4 (Supporting Information). For gene deletion, the *KanMX4* deletion cassette was amplified from the plasmid pROS13^[^ ^46^ ^]^ with primers containing a 41 bp homology arm and a 18 bp template matching sequence. The GTR‐CRISPR system was used for gene deletion or gene insertion. The guide RNA was designed through the following website: http://crispor.tefor.net/. Plasmid construction in the GTR‐CRISPR system was described in a previous study.^[^ ^44^ ^]^ The donor DNA contained 50 bp homology fragments upstream and downstream of the targets at both ends and was obtained by PCR. Yeast transformation was achieved using the LiAc/PEG method.^[^ ^47^ ^]^

### HPLC Analysis

For quantification of metabolites (glucose, ethanol, glycerol, etc.), strains were cultivated in SD‐2 × SCAA medium at 30 °C and 200 rpm. At various time points, 1 mL of fermentation broth was collected and centrifuged at 10 000 × g for 5 min. The supernatant was filtered through a 0.22 µm membrane and analyzed using an HPLC system (Shimadzu Corporation, Japan) equipped with an Aminex HPX‐87H column (Bio‐Rad). The HPLC system was operated at 45 °C with a flow rate of 0.6 mL min^−1^ using 5 mM H_2_SO_4_ as the mobile phase.

For squalene quantification, after fermentation for 96 h in a tube with YTD medium at 30 °C and 200 rpm, 500 µL of fermentation broth was mixed with 500 µL of ethyl acetate in a 1.5 mL tube containing 0.7 g of glass beads. The cells were disrupted twice using a Bioprep‐24R homogenizer (Allsheng) at 4 °C and 7 m s^−1^ for 1 min with an interval of 2 min. The mixture was then centrifuged, and the supernatant was filtered through a 0.22 µm syringe filter. An HPLC system (Shimadzu) equipped with a diode array detector and a Hypers11 ODS C18 column (Thermo Fisher Scientific) was used for product detection at 210 nm with acetonitrile as the mobile phase at a flow rate of 1.0 mL min^−1^.

For DHK quantification, after fermentation for 96 h in a tube containing YPD medium supplemented with 200 mg L⁻^1^ NAR at 30 °C and 200 rpm, 500 µL of fermentation broth was mixed with 500 µL of methanol for 5 min and then centrifuged at 10 000 × g for 5 min. Subsequently, the extracts were filtered through a 0.22 µm membrane and analyzed using an HPLC system equipped with a C18 column (Thermo Fisher Scientific). The following gradient elution method was used at a flow rate of 1.0 mL min^−1^: (A) 0.1% (v/v) TFA in acetonitrile, (B) 0.1% (v/v) trifluoroacetic acid (TFA) in water; 0−9 min 90−40% B, 9−19 min 40−60% B, 19−21 min 60−90% B, and 21−24 min 90% B. DHK was detected at a wavelength of 290 nm with retention times of ≈10.5 min.

### Oxygen Consumption Rate Assay

The OCR was measured with minor modifications from a previously described method.^[^ ^31^ ^]^ Strains were cultivated in SD‐2 × SCAA(+ura) at 30 °C and 200 rpm. Cells were harvested during the exponential growth phase (OD_600_ = 1‐1.5). 50 µL of medium containing 5 × 10^5^ cells was inoculated into a precoated Poly‐D Lysine (50 µg mL^−1^) XF 96‐well microplate. After incubating at 30°C for 30 min, 130 µL of medium was added to each well. The plate was left for another 30 min before the OCR was measured with a Seahorse XF Pro Analyzer (Agilent) according to the manufacturer's recommendations.

### qPCR

Yeast strains were cultivated in SD‐2 × SCAA(+ura) at 30°C and 200 rpm. The cells were harvested at an OD_600_ of 1‐1.5 and washed twice with PBS. RNA extraction was performed using a column yeast total RNA extraction and purification kit (Sangon Biotech). cDNA was reverse transcribed from mRNA by using the PrimeScript RT reagent Kit with gDNA Eraser (Takara). qPCR was performed with the SYBR Premix Ex Taq II kit (Takara) in a CFX96 Touch System (Bio‐Rad). The *ACT1* gene was used as a reference gene.

### Transcriptome Analysis

Strains L01, L02, and L03 and the control were cultivated in YTDP at 30°C and 200 rpm and used for transcriptome analysis. Yeast cells were collected from culture when the cell density reached OD_600_ = 1‐1.5 and were subsequently washed with sterile water. The cell pellets were stored in RNA‐Be‐Locker A reagent (Sangon Biotech, B644171). Library preparation, sequencing and data initial analysis were performed by Sangon Biotech (Shanghai, China). The raw data can be downloaded from the European Nucleotide Archive with access number PRJEB71089. Differential gene expression (DEG) was performed using the R package DEseq2. The R package Platform for Integrative Analysis of Omics (PIANO)^[^ ^48^ ^]^ was used to conduct reporter GO terms analysis, and the top five GO terms were selected based on the significance of up‐ or downregulation in distinct‐directional class.^[^ ^30^ ^]^

### Targeted Metabolomic Analysis

Strains L01, L02, and L03 and the control were cultivated in YTDP at 30°C and 200 rpm and used for metabolomic analysis. Yeast cells were collected at an OD_600_ of 1‐1.5, and the samples were subsequently freeze‐dried. An aliquot of each individual sample was precisely weighed and transferred to a tube. After adding two small steel balls and 500 µL of MeOH/H_2_O (3/1, v/v, precooled at ‐40°C), the samples were vortexed for 30 s, homogenized for 4 min at 35 Hz, and sonicated for 5 min in an ice‐water bath. This homogenization and sonication cycle was repeated three times, followed by incubation at ‐40°C for 1 h and centrifugation at 13 800 × g and 4°C for 15 min. The supernatants (400 µL) were collected and dried by centrifugation. The residues were reconstituted in 200 µL of water, vortexed, and filtered through a membrane filter. Finally, the sample was used for HPIC‐MRM‐MS analysis (Personalbio Biotechnology Co., Ltd., Shanghai, China).

Metabolomic profiling was conducted using a high‐performance ion chromatography system (HPIC, Thermo Scientific) equipped with Dionex IonPac AS11‐HC (2 × 250 mm) and AG11‐HC (2 mm × 50 mm) columns. Mobile phase A was 100 mM NaOH in water, while mobile phase D was ultrapure water. Another pumping system was used to supply the solvent (2 mM acetic acid in methanol) and the solvent mixed with the effluent before entering the electrospray ionization (ESI) (a flow rate of 0.15 mL min^−1^). The column temperature was set at 30°C. The autosampler temperature was set at 4°C, and the injection volume was 5 µL. An AB SCIEX 6500 QTRAP + triple quadrupole mass spectrometer equipped with an ESI interface was used for assay development. The typical ion source parameters included: IonSpray Voltage = ‐4 500 V, temperature = 450°C, Ion Source Gas 1 = 45 psi, Ion Source Gas 2 = 45 psi, and Curtain Gas = 30 psi.

### Protein Quantification

For α‐amylase quantification, strains were cultivated in SD‐2 × SCAA or YTDP media at 30°C and 200 rpm for 96 h. After fermentation, 500 µL of fermentation broth was collected and centrifuged at 10 000 × g for 5 min. The resulting supernatant was used for α‐amylase activity measurement via an α‐amylase assay kit (Megazyme, Ireland).

### ATP, NADH, and ROS Assays

For ATP, NADH, and ROS measurements, the strains were cultivated in YTDP medium at 30°C and 200 rpm. Yeast cells were collected from culture when the cell density reached OD_600_ = 1‐1.5. Then, the cell pellets were used for ATP, NADH, and ROS measurements by using BacTiter‐Glo Microbial Cell Viability Assay (Promega, USA), an NAD(H) assay kit (Cat No. BC0310, Solarbio, China) and DCFH‐DA (Solarbio, China), according to the manufacturer's recommendations.

### Fed‐Batch Fermentation

Fed‐batch fermentation for α‐amylase production was performed in a 1.3 L bioreactor system (Shanghai T&J Bio‐engineering). The L76 strain was inoculated in 300 mL of SD‐2 × SCAA (Na_2_HPO_4_·12H_2_O and NaH_2_PO_4_·2H_2_O were replaced with 2 g L^−1^ KH_2_PO_4_) with an initial OD_600_ = 0.1. The initial agitation speed was set at 600 rpm and was gradually increased to 1 200 rpm, which controlled the dissolved oxygen level above 30% in the bioreactor. The temperature was maintained at 30°C, and the pH was maintained at 6 (maintained by using 4 M KOH and 2 M HCl) in the bioreactor. During fed‐batch cultivation, the cells were initially fed low‐glucose feed medium (200 g L^−1^ glucose, 34.5 g L^−1^ yeast nitrogen base without amino acids, 25 g L^−1^ (NH4)_2_SO_4_, 50 g L^−1^ casamino acids (Formedium), 20 g L^−1^ KH_2_PO_4_, 50 mg L^−1^ uracil) to maintain a specific rate of 0.08 h^−1^. The high‐glucose feed medium contained 600 g L^−1^ glucose, with all other components being the same as those in the low‐glucose feed medium. After 60 h of cultivation, the high‐glucose medium feeding began when the dissolved oxygen level exceeded 25%.^[^ ^12^ ^]^ In total, 170 mL of low‐glucose feed medium and ≈110 mL of high‐glucose feed medium were added to the bioreactor. Biological experiments involving fed‐batch cultivation were conducted in triplicate.

### LincRNA Sequence Analysis

Genetic maps of lincRNAs were generated using a website (http://mg2c.iask.in/mg2c_v2.1/). The GC contents of the SUT and CUT sequences were analyzed to assess their compositional features. *S. cerevisiae* CEN.PK113‐7D genome information was retrieved from the NCBI database (https://www.ncbi.nlm.nih.gov/datasets/genome/GCA_002571405.2/). Minimum free energy predictions, indicating the stability of the optimal secondary structure of lincRNAs, were performed on a website (http://rna.tbi.univie.ac.at//cgi‐bin/RNAWebSuite/RNAfold.cgi). The minimum free energy of bases was calculated as: (minimum free energy of interest lincRNA)/(base number of interest lincRNA sequence) × 100%.

### Statistical Analysis

All experiments were conducted in duplicate or triplicate. A two‐tailed *t*‐test and analysis of variance were used for statistical analysis, which was performed with Microsoft Excel 2019. All the data are presented as mean ± SD, * marked as *P* < 0.05.

## Conflict of Interest

M.H., L.Q., Y.P., S.X., and C.X. applied for patents for the protection of strain development involving lincRNAs and their applications. The remaining authors declare no competing interests.

## Author Contributions

M.H. and L.Q. conceived and designed the research. L.Q., Y.P., S.X., Z.Y., C.X., and X.L. performed the research. L.Q., Y.P., D.Y., J.H., and M.H. analyzed the data. M.H. supervised the study. L.Q., Y.P., and M.H. wrote the manuscript; and all the authors read and approved the manuscript.

## Supporting information



Supporting Information

Supporting Information Table 1

## Data Availability

The data that support the findings of this study are available from the corresponding author upon reasonable request.
