# Supplementary material for: Multi‐Omics Analysis Reveals Impacts of LincRNA Deletion on Yeast Protein Synthesis
Source: Adv Sci (Weinh). 2025 Feb 14;12(13):2406873. doi: 10.1002/advs.202406873 (PMC11967807; doi:10.1002/advs.202406873)
Supplement: Supplementary file 1 — Supporting Information [file ADVS-12-2406873-s001.pdf]

ADVANCED SCIENCE
Open Access

Supporting Information

for *Adv. Sci.*, DOI 10.1002/advs.202406873

Multi-Omics Analysis Reveals Impacts of LincRNA Deletion on Yeast Protein Synthesis

*Ling Qin, Yuyang Pan, Songlyu Xue, Zhibo Yan, Chufan Xiao, Xiufang Liu, Dan Yuan, Jin Hou and Mingtao Huang**

Supporting information for

Multi-omics Analysis Reveals Impacts of LincRNA Deletion on Yeast Protein Synthesis

Ling Qin[1], Yuyang Pan[1], Songlyu Xue[1], Zhibo Yan[1], Chufan Xiao[1], Xiufang Liu[1], Dan Yuan[1], Jin Hou[2], and Mingtao Huang[1,*]

[1]School of Food Science and Engineering, South China University of Technology, Guangzhou, 510641, China

[2]State Key Laboratory of Microbial Technology, Shandong University, Qingdao, 266237, China

[*] To whom correspondence should be addressed. Email: huangmt@scut.edu.cn.

This file includes:

Figure S1 to Figure S14
Table S1 to Table S2

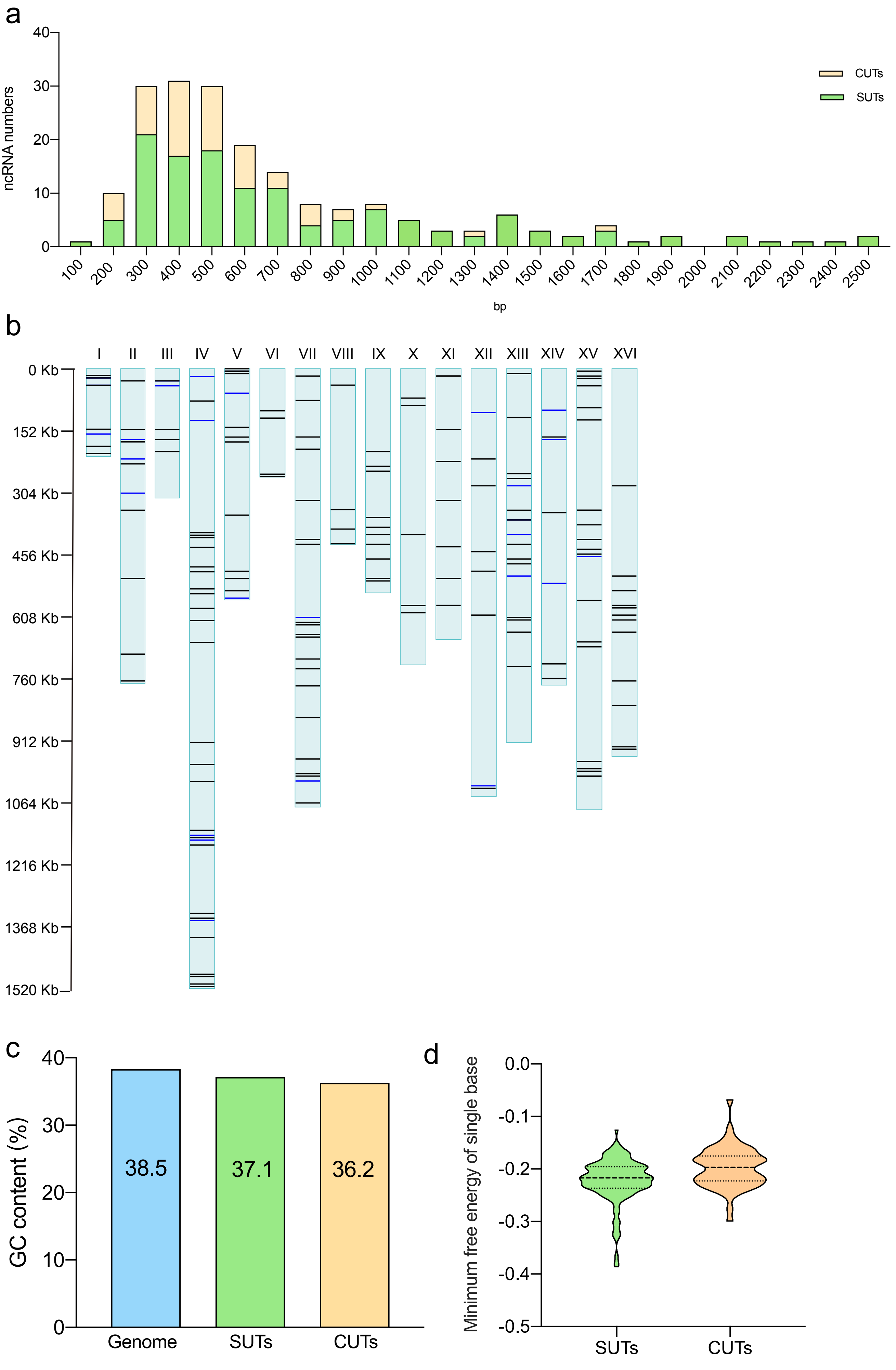

a
ncRNA numbers
40
30
20
10
0
CUTs
SUTs
100
200
300
400
500
600
700
800
900
1000
1100
1200
1300
1400
1500
1600
1700
1800
1900
2000
2100
2200
2300
2400
2500
bp
b
I
II
III
IV
V
VI
VII
VIII
IX
X
XI
XII
XIII
XIV
XV
XVI
0 Kb
152 Kb
304 Kb
456 Kb
608 Kb
760 Kb
912 Kb
1064 Kb
1216 Kb
1368 Kb
1520 Kb
c
GC content (%)
40
30
20
10
0
38.5
37.1
36.2
Genome
SUTs
CUTs
d
Minimum free energy of single base
0.0
-0.1
-0.2
-0.3
-0.4
-0.5
SUTs
CUTs

Figure S1. Characterization of LincRNAs. a) The length distribution of lincRNAs (SUTs and CUTs). b) The distribution of SUTs and CUTs across *S. cerevisiae* chromosomes. c) The GC base percentage in 132 SUTs and 55 CUTs sequences. d) The minimum free energy of a single base in SUTs and CUTs sequences.

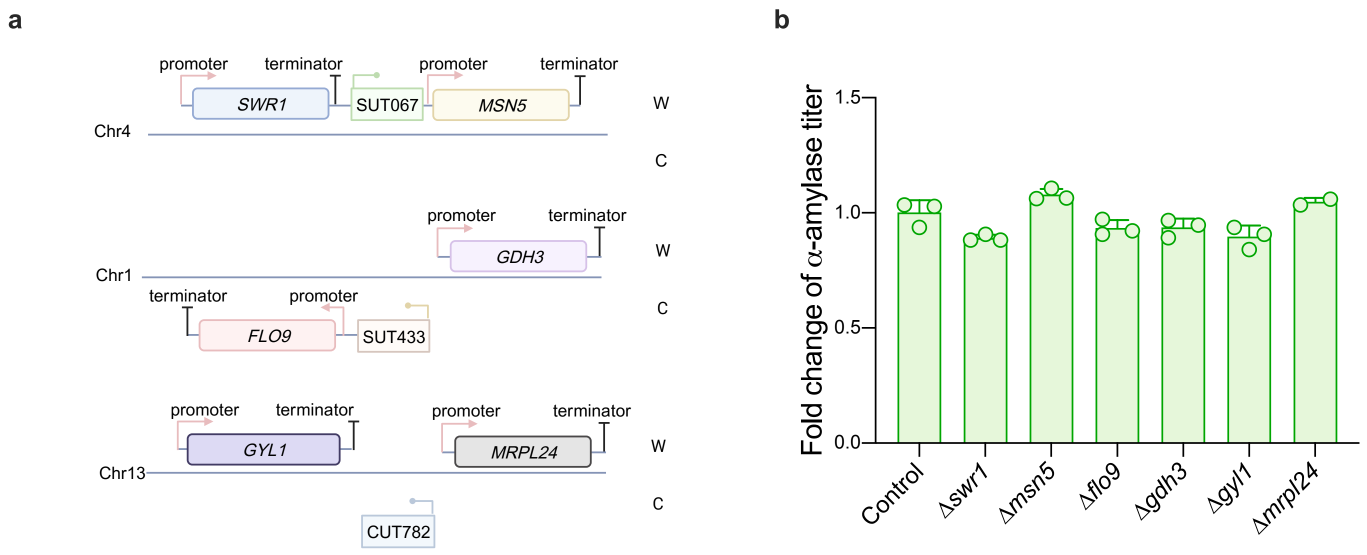

Figure S2. Impact of deletion of neighboring genes of target lincRNAs on α-amylase production. a) Neighboring genes of SUT067, SUT433, and CUT782. W: Watson strand; C: Crick strand. b) α-amylase titers of strains with deletions of neighboring genes. Strains were cultivated at 30 °C for 96 h for α-amylase production. Data shown are mean values ± SDs of biological duplicates or triplicates of single clones.

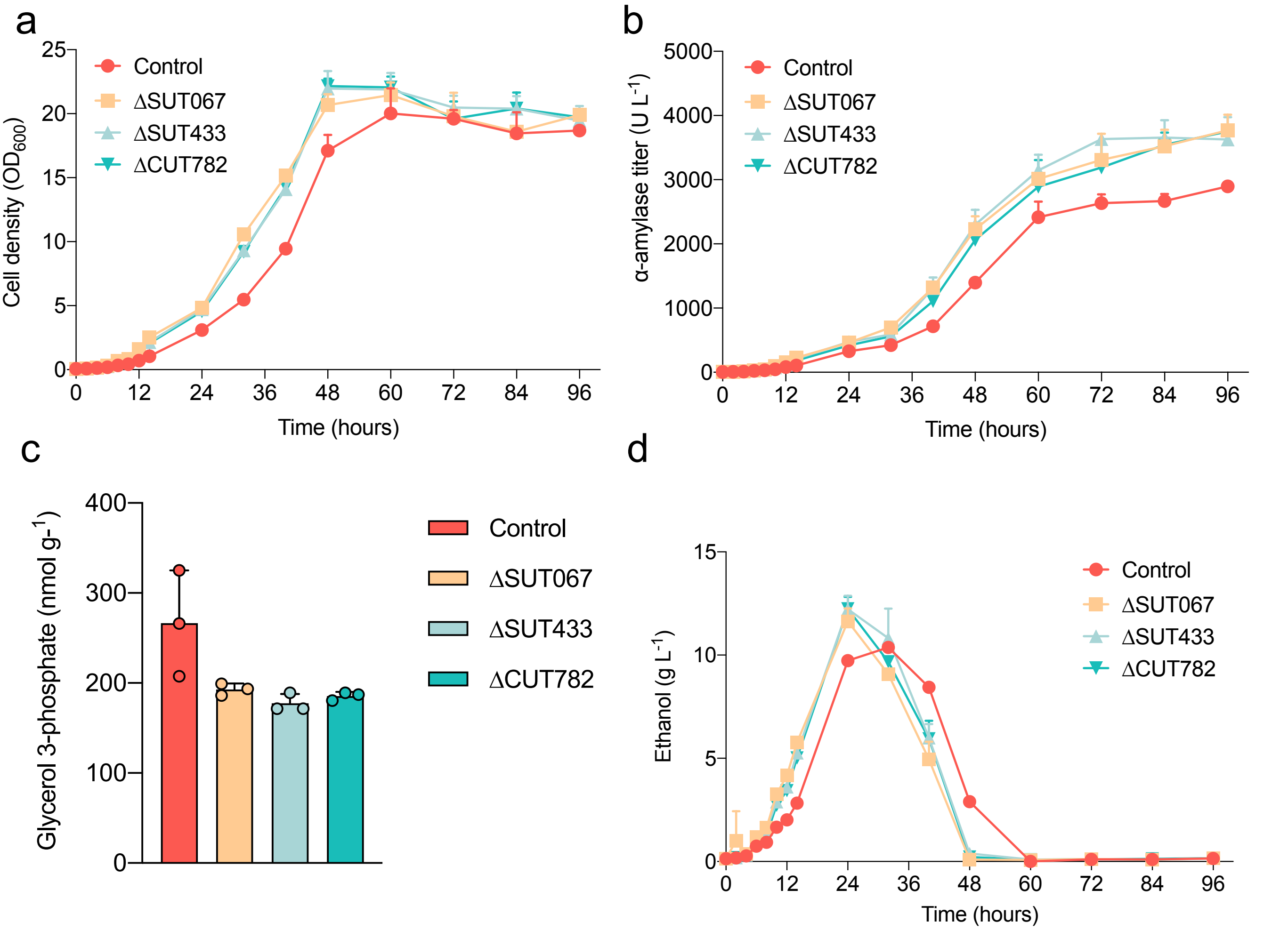

Figure S3. Batch cultivation of strains L01(ΔSUT067), L02(ΔSUT433) and L03(ΔCUT782) and the control strain (L0). a) Cell density. b) α-amylase titer. c) Glycerol-3-phosphate level. d) Ethanol production.

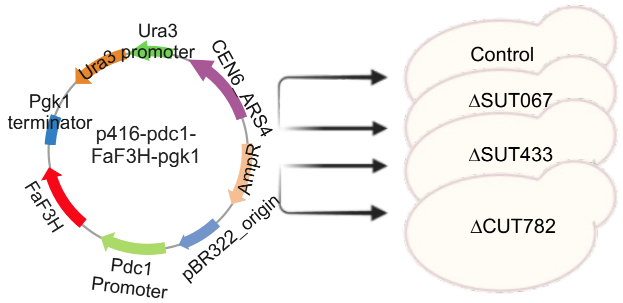

Figure S4. Schematic of expression plasmid construction. Overexpression of FaF3H by using the p416 plasmid for dihydrokaempferol production (created with BioRender.com).

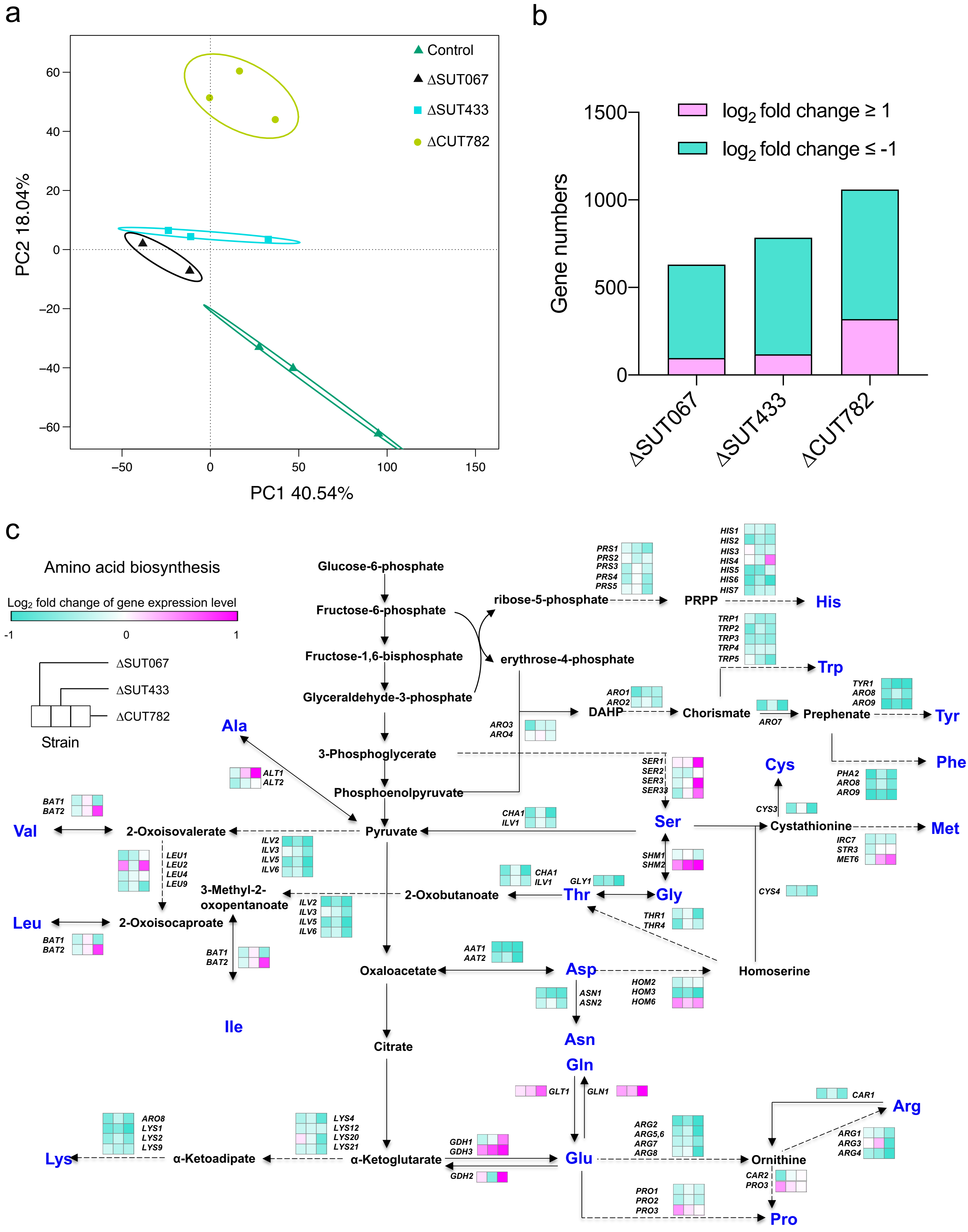
a
Control
ΔSUT067
ΔSUT433
ΔCUT782
PC2 18.04%
PC1 40.54%
60
40
20
0
−20
−40
−60
−50
0
50
100
150
b
log2 fold change ≥ 1
log2 fold change ≤ -1
Gene numbers
1500
1000
500
0
ΔSUT067
ΔSUT433
ΔCUT782
c
Amino acid biosynthesis
Log2 fold change of gene expression level
-1
0
1
ΔSUT067
ΔSUT433
ΔCUT782
Strain
Glucose-6-phosphate
Fructose-6-phosphate
Fructose-1,6-bisphosphate
Glyceraldehyde-3-phosphate
3-Phosphoglycerate
Phosphoenolpyruvate
Pyruvate
ribose-5-phosphate
erythrose-4-phosphate
PRPP
His
Trp
Tyr
Phe
DAHP
Chorismate
Prephenate
PRS1
PRS2
PRS3
PRS4
PRS5
HIS1
HIS2
HIS3
HIS4
HIS5
HIS6
HIS7
TRP1
TRP2
TRP3
TRP4
TRP5
ARO1
ARO2
ARO3
ARO4
ARO7
TYR1
ARO8
ARO9
PHA2
ARO8
ARO9
Ala
ALT1
ALT2
Val
BAT1
BAT2
2-Oxoisovalerate
LEU1
LEU2
LEU4
LEU9
Leu
2-Oxoisocaproate
BAT1
BAT2
3-Methyl-2-oxopentanoate
BAT1
BAT2
Ile
ILV2
ILV3
ILV5
ILV6
2-Oxobutanoate
CHA1
ILV1
Thr
GLY1
Gly
THR1
THR4
SER1
SER2
SER3
SER33
Ser
SHM1
SHM2
Cys
CYS3
Cystathionine
Met
IRC7
STR3
MET6
CYS4
Homoserine
HOM2
HOM3
HOM6
Oxaloacetate
AAT1
AAT2
Asp
ASN1
ASN2
Asn
Citrate
Gln
GLT1
GLN1
Glu
GDH1
GDH3
GDH2
α-Ketoglutarate
α-Ketoadipate
Lys
ARO8
LYS1
LYS2
LYS9
LYS4
LYS12
LYS20
LYS21
ARG2
ARG5,6
ARG7
ARG8
Ornithine
CAR1
Arg
ARG1
ARG3
ARG4
CAR2
PRO3
PRO1
PRO2
PRO3
Pro

Figure S5. Overview of transcriptome data. a) Principal component analysis. b) Differentially expressed genes (significantly DEGs, P-adj <0.05 and $|\log_2$ Fold change$| \geq 1$) in L01(ΔSUT067), L02(ΔSUT433) and L03(ΔCUT782) strains compared with the control L0. c) Transcriptional changes of genes involved in amino acid biosynthesis, L0 as control.

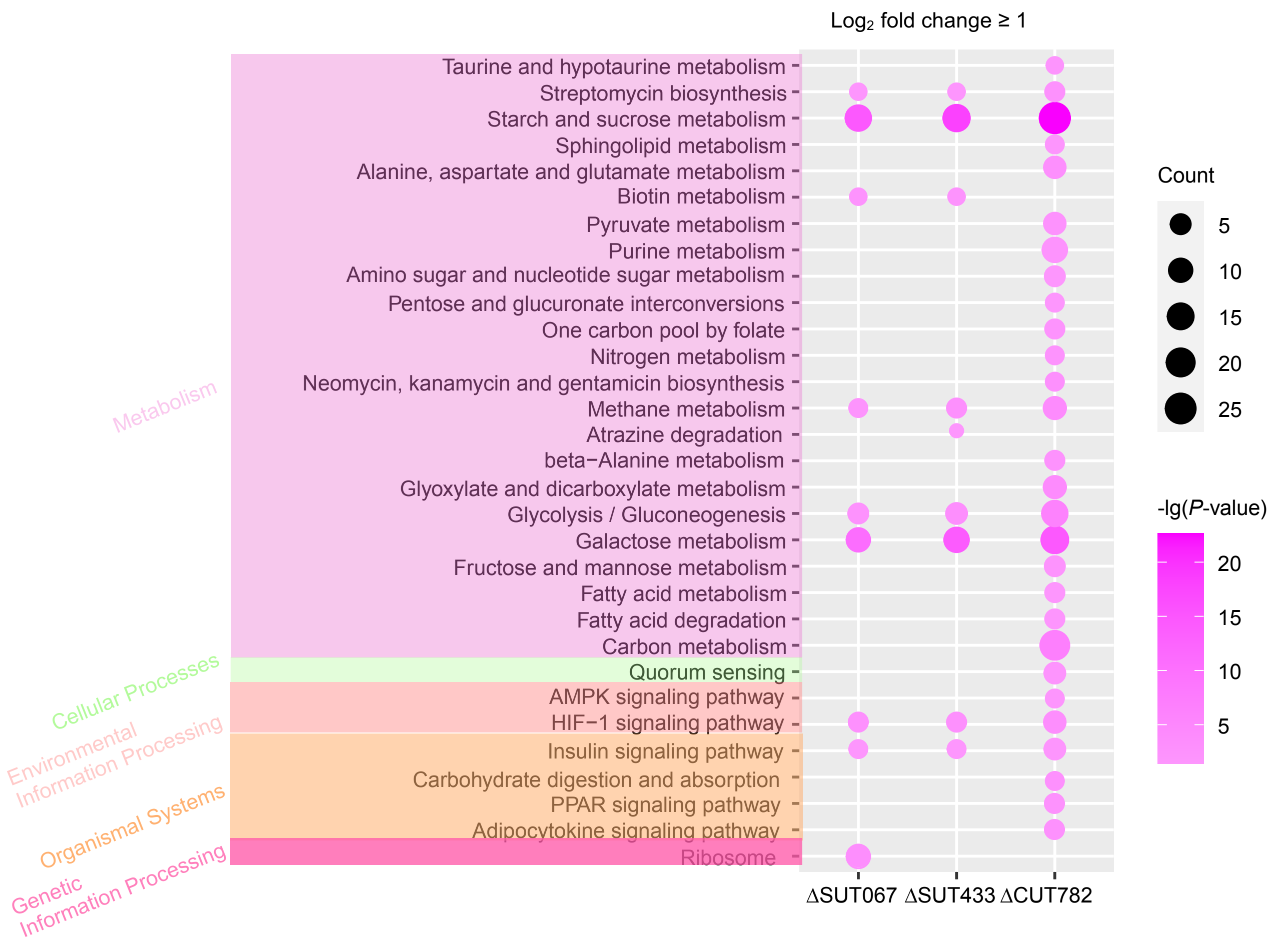

Figure S6. KEGG analysis. Upregulated DEGs were annotated by KEGG pathways ($\log_2$ fold change≥1).

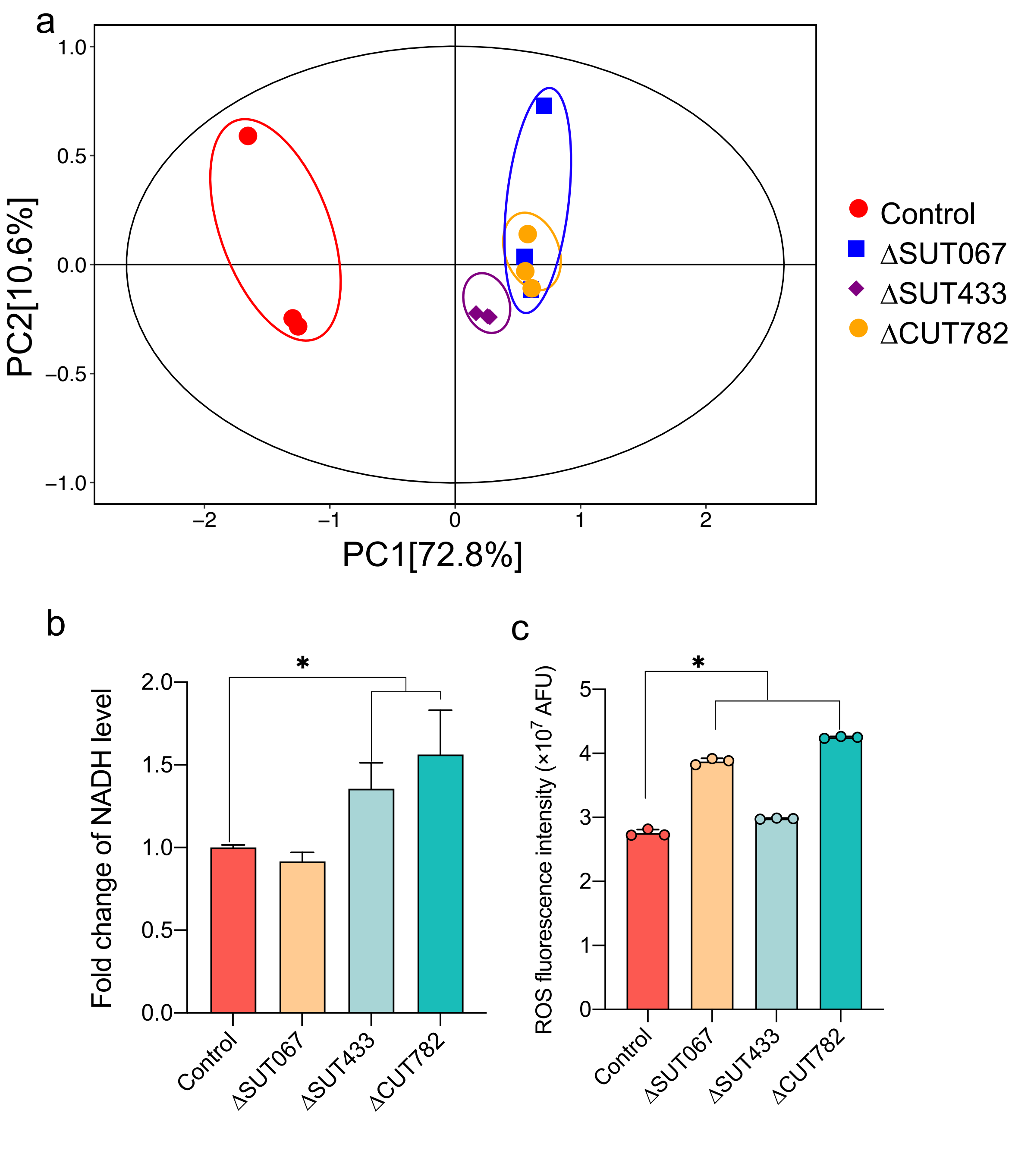

Figure S7. Overview of Metabolome data. a) Principal component analysis. b) Relative level of NADH in L01(ΔSUT067), L02(ΔSUT433) and L03(ΔCUT782) strains compared with the control L0. c) ROS levels in strains.

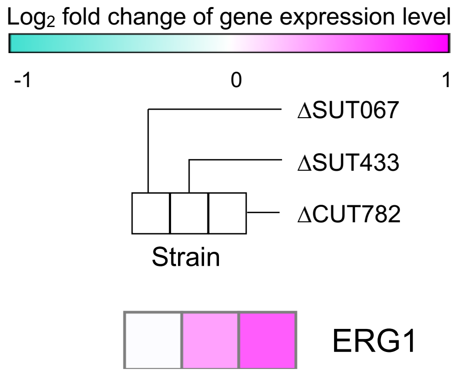

Figure S8. *ERG1* gene expression levels. *ERG1* expression levels in L01(ΔSUT067), L02(ΔSUT433) and L03(ΔCUT782) strains compared with the control L0.

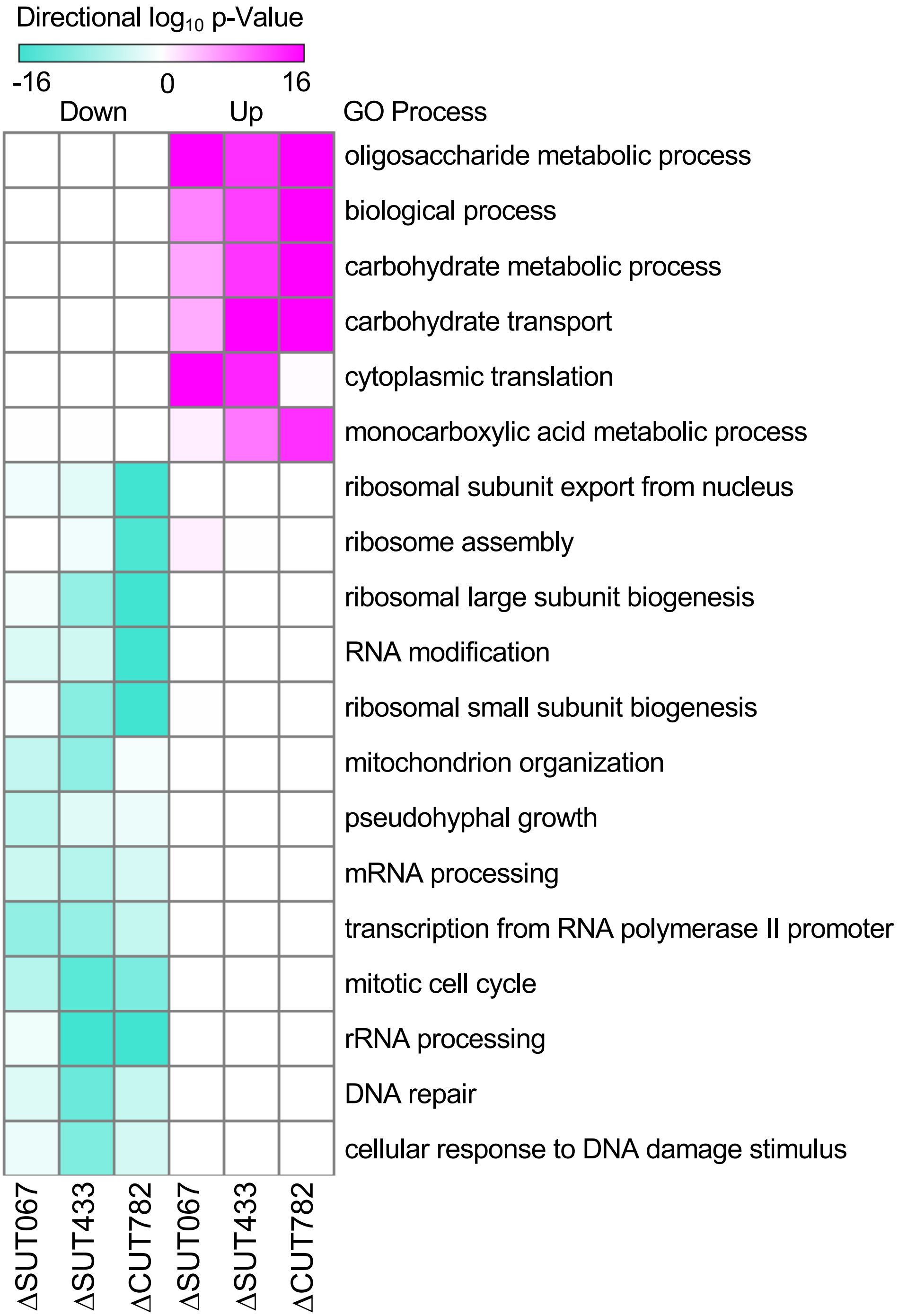

Figure S9. Transcriptional analysis of engineered strains. a) The five highest scoring GO terms for each strain in both the upregulated (marked in purple) and downregulated (marked in cyan) groups.

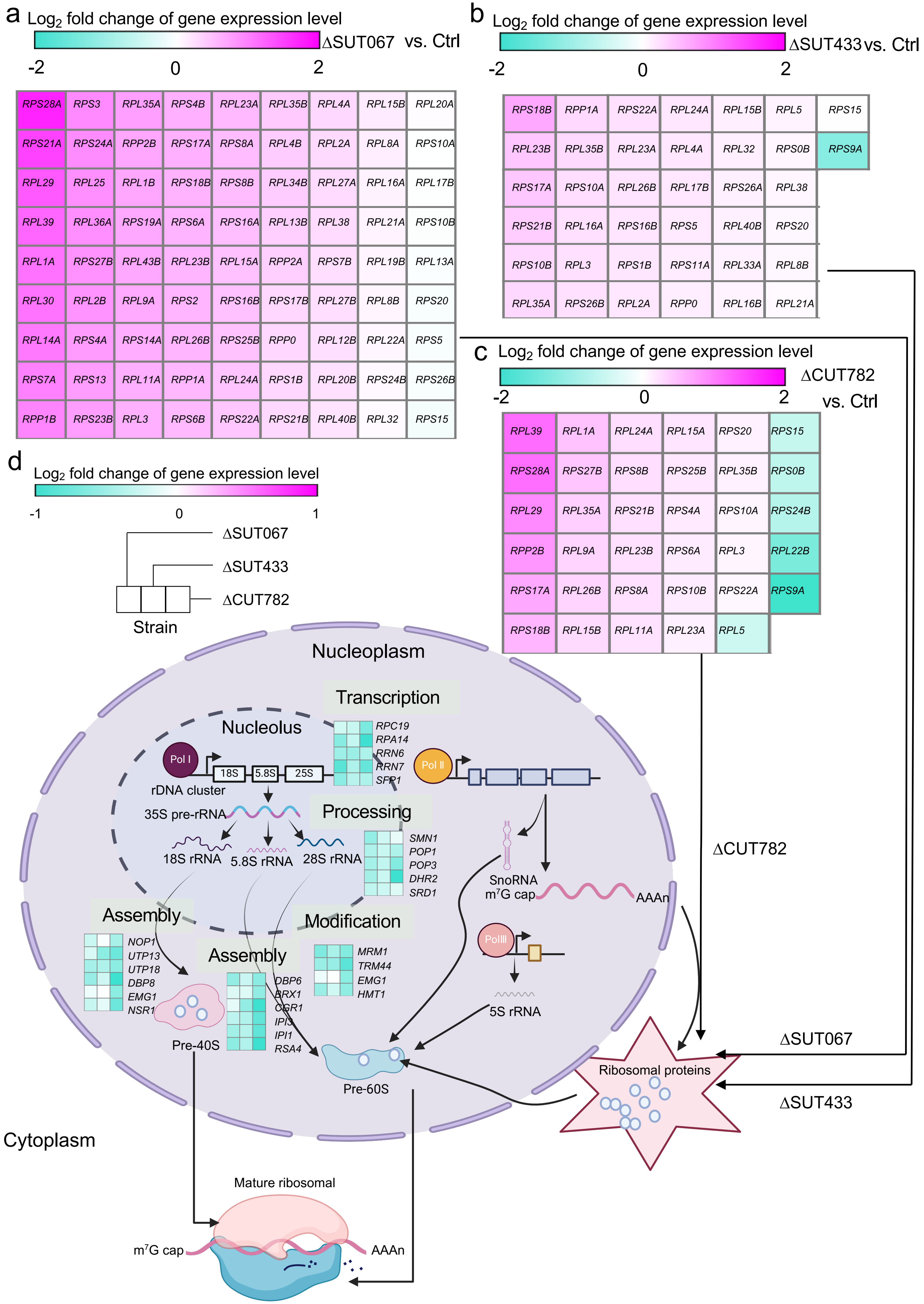

a
Log2 fold change of gene expression level
ΔSUT067 vs. Ctrl
-2
0
2
RPS28A RPS3 RPL35A RPS4B RPL23A RPL35B RPL4A RPL15B RPL20A
RPS21A RPS24A RPP2B RPS17A RPS8A RPL4B RPL2A RPL8A RPS10A
RPL29 RPL25 RPL1B RPS18B RPS8B RPL34B RPL27A RPL16A RPL17B
RPL39 RPL36A RPS19A RPS6A RPS16A RPL13B RPL38 RPL21A RPS10B
RPL1A RPS27B RPL43B RPL23B RPL15A RPP2A RPS7B RPL19B RPL13A
RPL30 RPL2B RPL9A RPS2 RPS16B RPS17B RPL27B RPL8B RPS20
RPL14A RPS4A RPS14A RPL26B RPS25B RPP0 RPL12B RPL22A RPS5
RPS7A RPS13 RPL11A RPP1A RPL24A RPS1B RPL20B RPS24B RPS26B
RPP1B RPS23B RPL3 RPS6B RPS22A RPS21B RPL40B RPL32 RPS15
b
Log2 fold change of gene expression level
ΔSUT433 vs. Ctrl
-2
0
2
RPS18B RPP1A RPS22A RPL24A RPL15B RPL5 RPS15
RPL23B RPL35B RPL23A RPL4A RPL32 RPS0B RPS9A
RPS17A RPS10A RPL26B RPL17B RPS26A RPL38
RPS21B RPL16A RPS16B RPS5 RPL40B RPS20
RPS10B RPL3 RPS1B RPS11A RPL33A RPL8B
RPL35A RPS26B RPL2A RPP0 RPL16B RPL21A
c
Log2 fold change of gene expression level
ΔCUT782
vs. Ctrl
-2
0
2
RPL39 RPL1A RPL24A RPL15A RPS20 RPS15
RPS28A RPS27B RPS8B RPS25B RPL35B RPS0B
RPL29 RPL35A RPS21B RPS4A RPS10A RPS24B
RPP2B RPL9A RPL23B RPS6A RPL3 RPL22B
RPS17A RPL26B RPS8A RPS10B RPS22A RPS9A
RPS18B RPL15B RPL11A RPL23A RPL5
d
Log2 fold change of gene expression level
-1
0
1
ΔSUT067
ΔSUT433
ΔCUT782
Strain
Nucleoplasm
Transcription
Nucleolus
RPC19
RPA14
RRN6
RRN7
SFP1
Pol I
rDNA cluster
18S
5.8S
25S
Pol II
35S pre-rRNA
Processing
18S rRNA
5.8S rRNA
28S rRNA
SMN1
POP1
POP3
DHR2
SRD1
SnoRNA
m7G cap
AAAn
ΔCUT782
Assembly
NOP1
UTP13
UTP18
DBP8
EMG1
NSR1
Modification
MRM1
TRM44
EMG1
HMT1
PolIII
5S rRNA
Assembly
DBP6
BRX1
CGR1
IPI3
IPI1
RSA4
Pre-40S
Pre-60S
Ribosomal proteins
ΔSUT067
ΔSUT433
Cytoplasm
Mature ribosomal
m7G cap
AAAn

Figure S10. Transcriptional analysis of differentially expressed genes. a) Transcriptional changes of genes involved in the biosynthesis of ribosomal proteins in strain L01(ΔSUT067) compared with the control L0. b) Transcriptional changes of genes involved in the biosynthesis of ribosomal proteins in strain L02(ΔSUT433) compared with the control L0. c) Transcriptional changes of genes involved in the biosynthesis of ribosomal proteins in strain L03(ΔCUT782) compared with the control L0 (created with BioRender.com). d) Transcriptional changes of genes involved in rRNA transcription, processing, modification, and assembly.

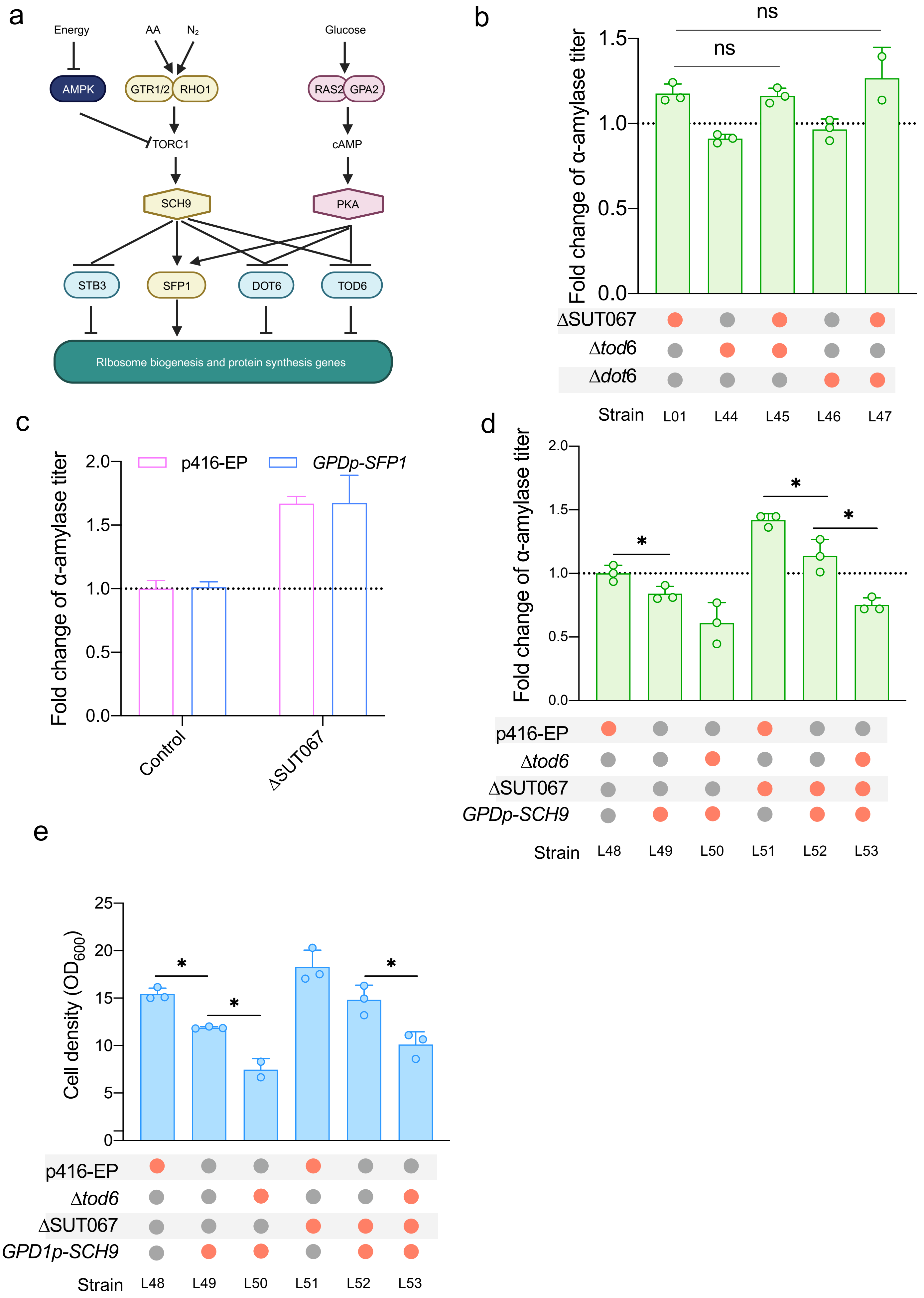

a
Energy
AA
N2
Glucose
AMPK
GTR1/2
RHO1
RAS2
GPA2
TORC1
cAMP
SCH9
PKA
STB3
SFP1
DOT6
TOD6
RIbosome biogenesis and protein synthesis genes
b
Fold change of α-amylase titer
ns
ns
ΔSUT067
Δtod6
Δdot6
Strain
L01
L44
L45
L46
L47
c
Fold change of α-amylase titer
p416-EP
GPDp-SFP1
Control
ΔSUT067
d
Fold change of α-amylase titer
p416-EP
Δtod6
ΔSUT067
GPDp-SCH9
Strain
L48
L49
L50
L51
L52
L53
e
Cell density (OD600)
p416-EP
Δtod6
ΔSUT067
GPD1p-SCH9
Strain
L48
L49
L50
L51
L52
L53

Figure S11. Engineering TORC1 and cAMP pathway genes to improve α-amylase secretion. a) A proposed model illustrating the function of the TORC1-PKA signaling circuit. b) α-amylase titers of engineered strains. c) α-amylase titers following overexpression of *SFP1* in strain L01(ΔSUT067) and the control strain L0. d) α-amylase titer of engineered strains. e) Cell density measurements. Strains were cultivated in SD-2×SCAA(+ura) medium at 30 °C for 96 h for α-amylase production, data shown are mean values ± SDs of biological triplicates of single clones. The statistical significance was determined by a two-tailed homoscedastic (equal variance) t test and indicated with an asterisk if $P < 0.05$.

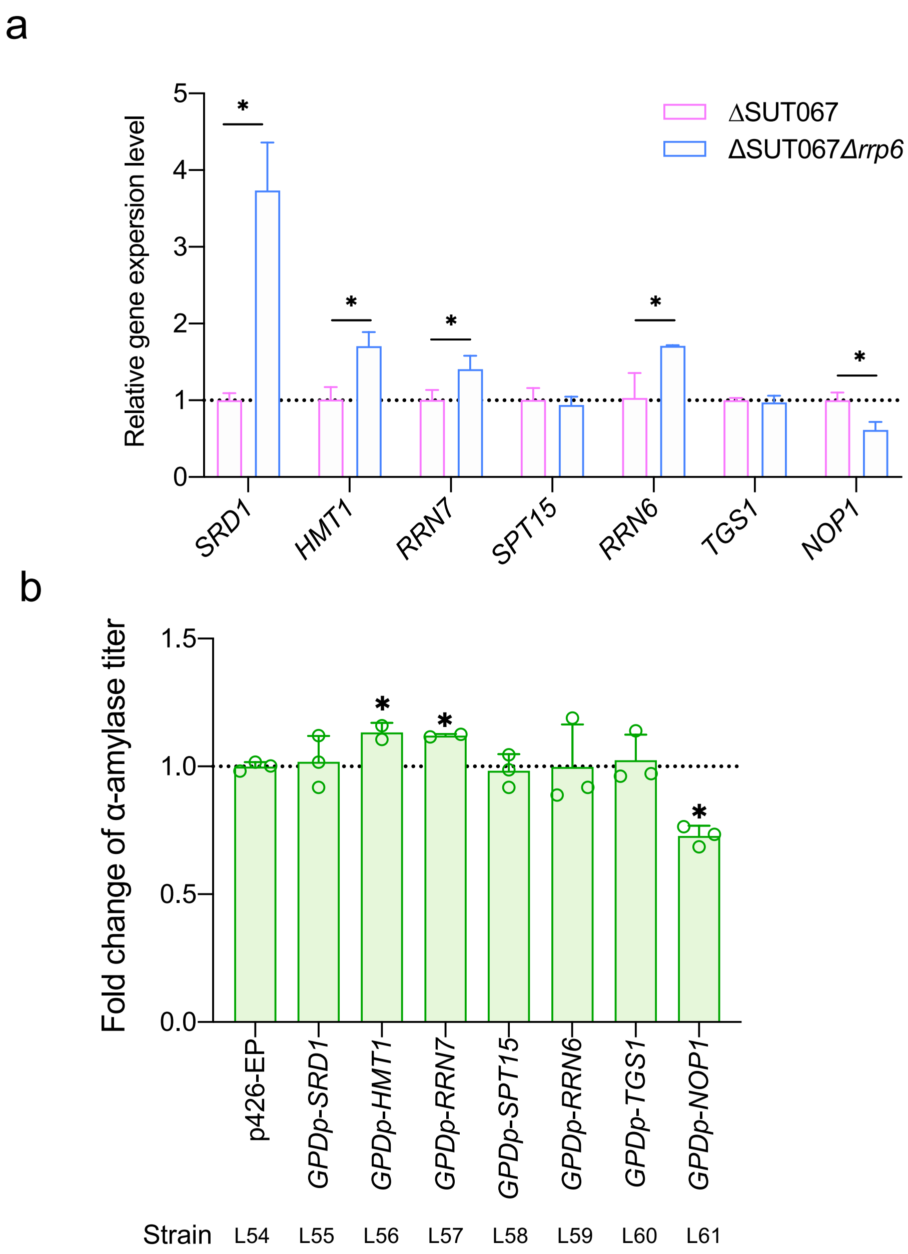

Figure S12. Deletion of *RRP6* gene enhances rRNA gene transcription. a) Expression levels of *SRD1*, *GMT1*, *RRN7*, *SPT15*, *RRN6*, *TGS1*, and *NOP1* genes in strain L41(LΔSUT067Δrrp6), strain L01 (ΔSUT067) as the reference. b) α-amylase titers following the overexpression of *SRD1*, *GMT1*, *RRN7*, *SPT15*, *RRN6*, *TGS1*, and *NOP1* genes in the starting strain L0, L54 with an empty plasmid as the reference strain. Strains were cultivated in SD-2×SCAA(+ura) medium at 30 °C for 96 h data shown are mean values ± SDs of biological triplicates. The statistical significance was determined by a two-tailed homoscedastic (equal variance) t test and indicated with an asterisk if $P < 0.05$.

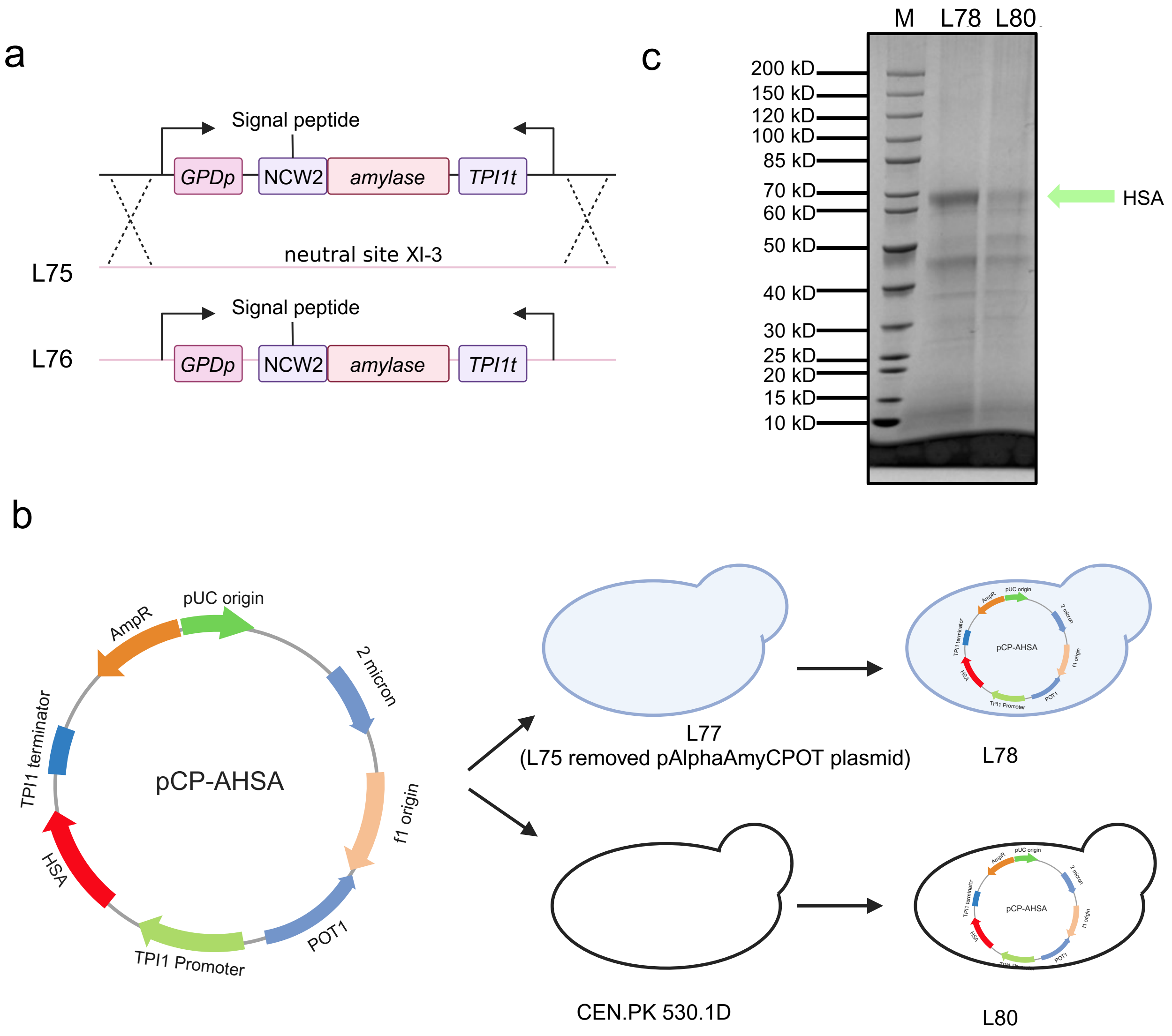

Figure S13. Increased secretion of human serum albumin in the engineered strain L78. a) A schematic detailing the construction of strain L76. b) Schematic representation of the construction of strains L78 and L80 (created with BioRender.com). c) SDS/PAGE analysis of the supernatant of engineered strains, M: Marker. Strains were cultivated in SD-2×SCAA(+ura, w/o BSA) medium at 30 °C for 96 h, data shown are mean values ± SDs of biological triplicates. The statistical significance was determined by a two-tailed homoscedastic (equal variance) t test and indicated with an asterisk if $P < 0.05$.

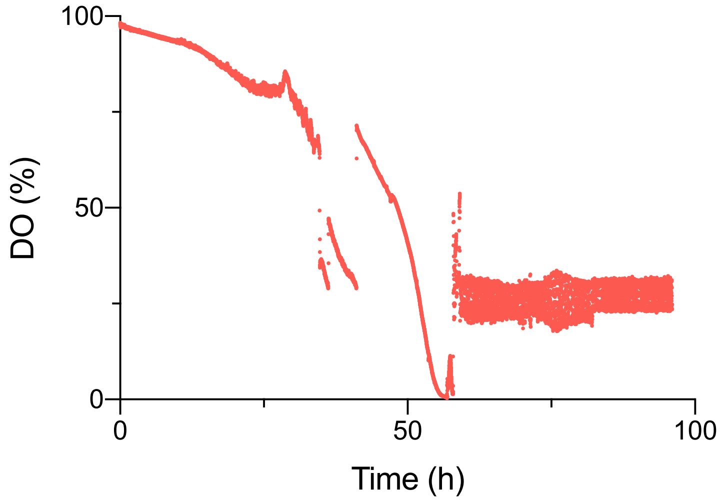

Figure S14. Dissolved oxygen level in fed-batch cultivation of strain L76.

Table S1. ***S. cerevisiae*** **strains used in this study.**

Strain No.	Genotype / containing plasmid	Reference
CEN.PK 530-1CK	*MATa URA3 tpi1(41-707)*	(Huang et al., 2018)[1]
AACK	*MATa URA3 tpi1(41-707)*; pAlphaAmyCPOT	This study
KΔSUT174	AACK; ΔSUT174::*KanMX4*	This study
KΔSUT456	AACK; ΔSUT456::*KanMX4*	This study
KΔSUT035	AACK; ΔSUT035::*KanMX4*	This study
KΔSUT414	AACK; ΔSUT414::*KanMX4*	This study
KΔSUT398	AACK; ΔSUT398::*KanMX4*	This study
KΔSUT010	AACK; ΔSUT010::*KanMX4*	This study
KΔSUT218	AACK; ΔSUT218::*KanMX4*	This study
KΔSUT532	AACK; ΔSUT532::*KanMX4*	This study
KΔSUT259	AACK; ΔSUT259::*KanMX4*	This study
KΔSUT167	AACK; ΔSUT167::*KanMX4*	This study
KΔSUT361	AACK; ΔSUT361::*KanMX4*	This study
KΔSUT553	AACK; ΔSUT553::*KanMX4*	This study
KΔSUT457	AACK; ΔSUT457::*KanMX4*	This study
KΔCUT060	AACK; ΔCUT060::*KanMX4*	This study
KΔCUT666	AACK; ΔCUT666::*KanMX4*	This study
KΔSUT298	AACK; ΔSUT298::*KanMX4*	This study
KΔSUT430	AACK; ΔSUT430::*KanMX4*	This study
KΔncRPR1	AACK; ΔncRPR1::*KanMX4*	This study
KΔSUT126	AACK; ΔSUT126::*KanMX4*	This study
KΔSUT329	AACK; ΔSUT329::*KanMX4*	This study
KΔSUT431	AACK; ΔSUT431::*KanMX4*	This study

KΔSUT362	AACK; ΔSUT362::*KanMX4*	This study
KΔSUT089	AACK; ΔSUT089::*KanMX4*	This study
KΔSUT451	AACK; ΔSUT451::*KanMX4*	This study
KΔSUT798	AACK; ΔSUT798::*KanMX4*	This study
KΔSUT546	AACK; ΔSUT546::*KanMX4*	This study
KΔSUT725	AACK; ΔSUT725::*KanMX4*	This study
KΔSUT477	AACK; ΔSUT477::*KanMX4*	This study
KΔSUT142	AACK; ΔSUT142::*KanMX4*	This study
KΔSUT388	AACK; ΔSUT388::*KanMX4*	This study
KΔSUT409	AACK; ΔSUT409::*KanMX4*	This study
KΔSUT109	AACK; ΔSUT109::*KanMX4*	This study
KΔSUT385	AACK; ΔSUT385::*KanMX4*	This study
KΔSUT543	AACK; ΔSUT543::*KanMX4*	This study
KΔSUT305	AACK; ΔSUT305::*KanMX4*	This study
KΔSUT086	AACK; ΔSUT086::*KanMX4*	This study
KΔSUT056	AACK; ΔSUT056::*KanMX4*	This study
KΔSUT229	AACK; ΔSUT229::*KanMX4*	This study
KΔSUT437	AACK; ΔSUT437::*KanMX4*	This study
KΔSUT248	AACK; ΔSUT248::*KanMX4*	This study
KΔSUT500	AACK; ΔSUT500::*KanMX4*	This study
KΔSUT099	AACK; ΔSUT099::*KanMX4*	This study
KΔSUT492	AACK; ΔSUT492::*KanMX4*	This study
KΔSUT503	AACK; ΔSUT503::*KanMX4*	This study
KΔSUT509	AACK; ΔSUT509::*KanMX4*	This study
KΔSUT233	AACK; ΔSUT233::*KanMX4*	This study

KΔSUT471	AACK; ΔSUT471::*KanMX4*	This study
KΔSUT617	AACK; ΔSUT617::*KanMX4*	This study
KΔncNME1	AACK; ΔncNME1::*KanMX4*	This study
KΔncRUF22	AACK; ΔncRUF22::*KanMX4*	This study
KΔSUT602	AACK; ΔSUT602::*KanMX4*	This study
KΔSUT540	AACK; ΔSUT540::*KanMX4*	This study
KΔSUT764	AACK; ΔSUT764::*KanMX4*	This study
KΔSUT501	AACK; ΔSUT501::*KanMX4*	This study
KΔSUT311	AACK; ΔSUT311::*KanMX4*	This study
KΔSUT830	AACK; ΔSUT830::*KanMX4*	This study
KΔSUT285	AACK; ΔSUT285::*KanMX4*	This study
KΔSUT289	AACK; ΔSUT289::*KanMX4*	This study
KΔSUT737	AACK; ΔSUT737::*KanMX4*	This study
KΔSUT591	AACK; ΔSUT591::*KanMX4*	This study
KΔSUT570	AACK; ΔSUT570::*KanMX4*	This study
KΔSUT581	AACK; ΔSUT581::*KanMX4*	This study
KΔSUT480	AACK; ΔSUT480::*KanMX4*	This study
KΔncTLC1	AACK; ΔncTLC1::*KanMX4*	This study
KΔncSCR1	AACK; ΔncSCR1::*KanMX4*	This study
KΔSUT571	AACK; ΔSUT571::*KanMX4*	This study
KΔSUT824	AACK; ΔSUT824::*KanMX4*	This study
KΔSUT593	AACK; ΔSUT593::*KanMX4*	This study
KΔSUT001	AACK; ΔSUT001::*KanMX4*	This study
KΔSUT496	AACK; ΔSUT496::*KanMX4*	This study
KΔSUT463	AACK; ΔSUT463::*KanMX4*	This study

KΔSUT796	AACK; ΔSUT796::*KanMX4*	This study
KΔSUT007	AACK; ΔSUT007::*KanMX4*	This study
KΔSUT353	AACK; ΔSUT353::*KanMX4*	This study
KΔSUT049	AACK; ΔSUT049::*KanMX4*	This study
KΔSUT834	AACK; ΔSUT834::*KanMX4*	This study
KΔSUT125	AACK; ΔSUT125::*KanMX4*	This study
KΔSUT075	AACK; ΔSUT075::*KanMX4*	This study
KΔSUT107	AACK; ΔSUT107::*KanMX4*	This study
KΔSUT304	AACK; ΔSUT304::*KanMX4*	This study
KΔSUT004	AACK; ΔSUT004::*KanMX4*	This study
KΔSUT067	AACK; ΔSUT067::*KanMX4*	This study
KΔSUT014	AACK; ΔSUT014::*KanMX4*	This study
KΔSUT019	AACK; ΔSUT019::*KanMX4*	This study
KΔSUT842	AACK; ΔSUT842::*KanMX4*	This study
KΔSUT493	AACK; ΔSUT493::*KanMX4*	This study
KΔSUT433	AACK; ΔSUT433::*KanMX4*	This study
KΔSUT847	AACK; ΔSUT847::*KanMX4*	This study
KΔSUT461	AACK; ΔSUT461::*KanMX4*	This study
KΔSUT413	AACK; ΔSUT413::*KanMX4*	This study
KΔSUT722	AACK; ΔSUT722::*KanMX4*	This study
KΔSUT441	AACK; ΔSUT441::*KanMX4*	This study
KΔSUT827	AACK; ΔSUT827::*KanMX4*	This study
KΔSUT432	AACK; ΔSUT432::*KanMX4*	This study
KΔSUT672	AACK; ΔSUT672::*KanMX4*	This study
KΔSUT779	AACK; ΔSUT779::*KanMX4*	This study

KΔSUT733	AACK; ΔSUT733::*KanMX4*	This study
KΔSUT249	AACK; ΔSUT249::*KanMX4*	This study
KΔSUT119	AACK; ΔSUT119::*KanMX4*	This study
KΔSUT084	AACK; ΔSUT084::*KanMX4*	This study
KΔSUT473	AACK; ΔSUT473::*KanMX4*	This study
KΔSUT347	AACK; ΔSUT347::*KanMX4*	This study
KΔSUT239	AACK; ΔSUT239::*KanMX4*	This study
KΔSUT376	AACK; ΔSUT376::*KanMX4*	This study
KΔSUT469	AACK; ΔSUT469::*KanMX4*	This study
KΔSUT083	AACK; ΔSUT083::*KanMX4*	This study
KΔSUT053	AACK; ΔSUT053::*KanMX4*	This study
KΔSUT367	AACK; ΔSUT367::*KanMX4*	This study
KΔSUT346	AACK; ΔSUT346::*KanMX4*	This study
KΔSUT219	AACK; ΔSUT219::*KanMX4*	This study
KΔSUT135	AACK; ΔSUT135::*KanMX4*	This study
KΔSUT157	AACK; ΔSUT157::*KanMX4*	This study
KΔSUT420	AACK; ΔSUT420::*KanMX4*	This study
KΔSUT274	AACK; ΔSUT274::*KanMX4*	This study
KΔSUT349	AACK; ΔSUT349::*KanMX4*	This study
KΔSUT087	AACK; ΔSUT087::*KanMX4*	This study
KΔSUT085	AACK; ΔSUT085::*KanMX4*	This study
KΔSUT211	AACK; ΔSUT211::*KanMX4*	This study
KΔSUT417	AACK; ΔSUT417::*KanMX4*	This study
KΔSUT284	AACK; ΔSUT284::*KanMX4*	This study
KΔSUT264	AACK; ΔSUT264::*KanMX4*	This study

KΔSUT123	AACK; ΔSUT123::*KanMX4*	This study
KΔSUT539	AACK; ΔSUT539::*KanMX4*	This study
KΔSUT418	AACK; ΔSUT418::*KanMX4*	This study
KΔSUT370	AACK; ΔSUT370::*KanMX4*	This study
KΔSUT355	AACK; ΔSUT355::*KanMX4*	This study
KΔSUT234	AACK; ΔSUT234::*KanMX4*	This study
KΔSUT518	AACK; ΔSUT518::*KanMX4*	This study
KΔSUT042	AACK; ΔSUT042::*KanMX4*	This study
KΔSUT173	AACK; ΔSUT173::*KanMX4*	This study
KΔSUT467	AACK; ΔSUT467::*KanMX4*	This study
KΔSUT111	AACK; ΔSUT111::*KanMX4*	This study
KΔSUT193	AACK; ΔSUT193::*KanMX4*	This study
KΔSUT479	AACK; ΔSUT479::*KanMX4*	This study
KΔSUT131	AACK; ΔSUT131::*KanMX4*	This study
KΔSUT616	AACK; ΔSUT616::*KanMX4*	This study
KΔCUT764	AACK; ΔCUT764::*KanMX4*	This study
KΔCUT816	AACK; ΔCUT816::*KanMX4*	This study
KΔCUT827	AACK; ΔCUT827::*KanMX4*	This study
KΔSUT643	AACK; ΔSUT643::*KanMX4*	This study
KΔCUT083	AACK; ΔCUT083::*KanMX4*	This study
KΔCUT782	AACK; ΔCUT782::*KanMX4*	This study
KΔCUT775	AACK; ΔCUT775::*KanMX4*	This study
KΔCUT296	AACK; ΔCUT296::*KanMX4*	This study
KΔCUT170	AACK; ΔCUT170::*KanMX4*	This study
KΔCUT015	AACK; ΔCUT015::*KanMX4*	This study

KΔCUT217	AACK; ΔCUT217::*KanMX4*	This study
KΔCUT001	AACK; ΔCUT001::*KanMX4*	This study
KΔSUT375	AACK; ΔSUT375::*KanMX4*	This study
KΔCUT645	AACK; ΔCUT645::*KanMX4*	This study
KΔncRUF21	AACK; ΔncRUF21::*KanMX4*	This study
KΔSUT339	AACK; ΔSUT339::*KanMX4*	This study
KΔCUT248	AACK; ΔCUT248::*KanMX4*	This study
KΔCUT103	AACK; ΔCUT103::*KanMX4*	This study
KΔCUT084	AACK; ΔCUT084::*KanMX4*	This study
KΔCUT238	AACK; ΔCUT238::*KanMX4*	This study
KΔCUT523	AACK; ΔCUT523::*KanMX4*	This study
KΔCUT436	AACK; ΔCUT436::*KanMX4*	This study
KΔCUT572	AACK; ΔCUT572::*KanMX4*	This study
KΔCUT379	AACK; ΔCUT379::*KanMX4*	This study
KΔCUT378	AACK; ΔCUT378::*KanMX4*	This study
KΔCUT441	AACK; ΔCUT441::*KanMX4*	This study
KΔCUT320	AACK; ΔCUT320::*KanMX4*	This study
KΔCUT244	AACK; ΔCUT244::*KanMX4*	This study
KΔCUT002	AACK; ΔCUT002::*KanMX4*	This study
KΔCUT142	AACK; ΔCUT142::*KanMX4*	This study
KΔCUT058	AACK; ΔCUT058::*KanMX4*	This study
KΔCUT007	AACK; ΔCUT007::*KanMX4*	This study
KΔCUT008	AACK; ΔCUT008::*KanMX4*	This study
KΔCUT277	AACK; ΔCUT277::*KanMX4*	This study
KΔCUT040	AACK; ΔCUT040::*KanMX4*	This study

KΔCUT005	AACK; ΔCUT005::*KanMX4*	This study
KΔCUT332	AACK; ΔCUT332::*KanMX4*	This study
KΔCUT315	AACK; ΔCUT315::*KanMX4*	This study
KΔCUT634	AACK; ΔCUT634::*KanMX4*	This study
KΔCUT547	AACK; ΔCUT547::*KanMX4*	This study
KΔCUT465	AACK; ΔCUT465::*KanMX4*	This study
KΔCUT575	AACK; ΔCUT575::*KanMX4*	This study
KΔCUT468	AACK; ΔCUT468::*KanMX4*	This study
KΔCUT458	AACK; ΔCUT458::*KanMX4*	This study
KΔCUT541	AACK; ΔCUT541::*KanMX4*	This study
KΔCUT546	AACK; ΔCUT546::*KanMX4*	This study
KΔCUT424	AACK; ΔCUT424::*KanMX4*	This study
KΔCUT356	AACK; ΔCUT356::*KanMX4*	This study
KΔCUT374	AACK; ΔCUT374::*KanMX4*	This study
KΔCUT462	AACK; ΔCUT462::*KanMX4*	This study
KΔCUT095	AACK; ΔCUT095::*KanMX4*	This study
KΔCUT474	AACK; ΔCUT474::*KanMX4*	This study
KΔCUT338	AACK; ΔCUT338::*KanMX4*	This study
KΔSUT348	AACK; ΔSUT348::*KanMX4*	This study
KΔSUT729	AACK; ΔSUT729::*KanMX4*	This study
KΔSUT098	AACK; ΔSUT098::*KanMX4*	This study
KΔSUT515	AACK; ΔSUT515::*KanMX4*	This study
KΔCUT123	AACK; ΔCUT123::*KanMX4*	This study
KΔSUT055	AACK; ΔSUT055::*KanMX4*	This study
KΔSUT267	AACK; ΔSUT267::*KanMX4*	This study

CEN.PK 530-1D	*MATa ura3-52 tpi1(41-707)::loxP-KanMX4-loxP*	(Huang et al., 2018)[1]
L0	CEN.PK 530.1D; pAlphaAmyCPOT; starter strain serving as control	This study
L01	CEN.PK 530.1D; pAlphaAmyCPOT; ΔSUT067	This study
L02	CEN.PK 530.1D; pAlphaAmyCPOT; ΔSUT433	This study
L03	CEN.PK 530.1D; pAlphaAmyCPOT; ΔCUT782	This study
L06	CEN.PK 530.1D; pAlphaAmyCPOT; ΔSUT067; ΔSUT591;ΔCUT378	This study
L07	CEN.PK 530.1D; pAlphaAmyCPOT; ΔSUT067; ΔCUT672	This study
L08	CEN.PK 530.1D; pAlphaAmyCPOT; ΔSUT067; ΔCUT782	This study
L09	CEN.PK 530.1D; pAlphaAmyCPOT; ΔSUT067; ΔCUT782;ΔSUT433	This study
L10	CEN.PK 530.1D; pAlphaAmyCPOT; ΔSUT067; ΔCUT462	This study
L11	CEN.PK 530.1D; pAlphaAmyCPOT; ΔSUT067; ΔSUT311	This study
L12	CEN.PK 530.1D; pAlphaAmyCPOT; ΔSUT067; ΔSUT126	This study
L13	CEN.PK 530.1D; pAlphaAmyCPOT; ΔSUT067; ΔCUT461	This study
L14	CEN.PK 530.1D; pAlphaAmyCPOT; ΔSUT067; ΔSUT496	This study
L15	CEN.PK 530.1D; pAlphaAmyCPOT; ΔSUT067; ΔCUT084	This study
L16	CEN.PK 530.1D; pAlphaAmyCPOT; ΔSUT067; ΔSUT457	This study
L17	CEN.PK 530.1D; pAlphaAmyCPOT; ΔSUT067; ΔSUT842	This study
L18	CEN.PK 530.1D; pAlphaAmyCPOT; ΔSUT067; ΔCUT378	This study
L19	CEN.PK 530.1D; pAlphaAmyCPOT; ΔSUT067; ΔSUT591	This study
L20	CEN.PK 530.1D; pAlphaAmyCPOT; ΔSUT067; ΔSUT591; ΔCUT084	This study
L21	CEN.PK 530.1D; pAlphaAmyCPOT; ΔSUT067; ΔSUT591; ΔSUT842	This study
L22	CEN.PK 530.1D; pAlphaAmyCPOT; ΔSUT067; ΔSUT591; ΔSUT496	This study
L23	CEN.PK 530.1D; pAlphaAmyCPOT; ΔSUT067; ΔSUT591; ΔSUT457	This study
L24	L0; p416-*PDC1p-FaF3H*	This study
L25	L01; p416-*PDC1p-FaF3H*	This study

L26	L02; p416-*PDC1p-FaF3H*	This study
L27	L03; p416-*PDC1p-FaF3H*	This study
L32	L0; p416-*GPDp-tHMG1*	This study
L33	L01; p416-*GPDp-tHMG1*	This study
L34	L02; p416-*GPDp-tHMG1*	This study
L35	L03; p416-*GPDp-tHMG1*	This study
L36	L0; XI-3::*GPDp-Ncw2-amylase*	This study
L37	L01; XI-3::*GPDp-Ncw2-amylase*	This study
L38	L02; XI-3::*GPDp-Ncw2-amylase*	This study
L39	L03; XI-3::*GPDp-Ncw2-amylase*	This study
L40	L0; Δ*rrp6*	This study
L41	L01; Δ*rrp6*	This study
L42	L02; Δ*rrp6*	This study
L43	L03; Δ*rrp6*	This study
L44	L0; Δ*tod6*::*BleR*	This study
L45	L01 Δ*tod6*::*BleR*	This study
L46	L0;Δ*dot*6::*BleR*	This study
L47	L01 Δ*dot*6::*BleR*	This study
L48	L0; p416-EP	This study
L49	L0; p416-*GPDp-SCH9*	This study
L50	L44; p416-*GPDp-SCH9*	This study
L51	L01; p416-EP	This study
L52	L01; p416-*GPDp-SCH9*	This study
L53	L45; p416-*GPDp-SCH9*	This study
L54	L0; p426-EP	This study

L55	L0; p426-GPDp-*SRD1*	This study
L56	L0; p426-*GPDp-HMT1*	This study
L57	L0; p426-*GPDp-RRN7*	This study
L58	L0; p426-*GPDp-SPT15*	This study
L59	L0; p426-*GPDp-RRN6*	This study
L60	L0; p426-*GPDp-TGS1*	This study
L61	L0; p426-*GPDp-NOP1*	This study
L62	L0; X-3::*GPDp-SSB1*	This study
L63	L01; X-3::*GPDp-SSB1*	This study
L64	L02; X-3::*GPDp-SSB1*	This study
L65	L03; X-3::*GPDp-SSB1*	This study
L66	L62; XI-3::*GPDp-Ncw2-amylase*	This study
L67	L62; Δ*sec72*	This study
L68	L67; Δ*rrp6*	This study
L69	L68; ΔSUT067	This study
L70	L68; ΔSUT433	This study
L71	L68; ΔCUT782	This study
L72	L68; ΔSUT433::*PGK1p-SIS1*	This study
L73	L72; Δ*der1*	This study
L74	L73; *KAR2p*::*GPDp*	This study
L75	L74; *PDI1p*::*TEF1p*	This study
L76	L75; XI-3::*GPDp-Ncw2-amylase*	This study
L77	CEN.PK 530.1D; X3::*GPDp-SSB1-CYC1t*; Δ*sec72*; Δ*rrp6*;ΔSUT433::<br>*PGK1p-SIS1*;Δ*der1*;*KAR2p*::*GPDp*;*PDI1p*::*TEF1p*	This study
L78	L77; pCP-AHSA	This study

L80	CEN.PK 530.1D;pCP-AHSA	This study
Q01	CEN.PK 530.1D; pAlphaAmyCPOT; ΔSUT457	This study
Q02	CEN.PK 530.1D; pAlphaAmyCPOT; ΔCUT378	This study
Q03	CEN.PK 530.1D; pAlphaAmyCPOT; ΔCUT672	This study
Q04	CEN.PK 530.1D; pAlphaAmyCPOT; ΔSUT311	This study
Q05	CEN.PK 530.1D; pAlphaAmyCPOT; ΔSUT493	This study
Q06	CEN.PK 530.1D; pAlphaAmyCPOT; ΔCUT084	This study
Q07	CEN.PK 530.1D; pAlphaAmyCPOT; ΔSUT842	This study
Q08	CEN.PK 530.1D; pAlphaAmyCPOT; ΔSUT126	This study
Q09	CEN.PK 530.1D; pAlphaAmyCPOT; ΔSUT218	This study
Q10	CEN.PK 530.1D; pAlphaAmyCPOT; ΔCUT462	This study
Q11	CEN.PK 530.1D; pAlphaAmyCPOT; ΔSUT362	This study
Q12	CEN.PK 530.1D; pAlphaAmyCPOT; ΔSUT089	This study
Q13	CEN.PK 530.1D; pAlphaAmyCPOT; ΔCUT461	This study
Q14	CEN.PK 530.1D; pAlphaAmyCPOT; ΔSUT007	This study
Q15	CEN.PK 530.1D; pAlphaAmyCPOT; ΔSUT398	This study
Q16	CEN.PK 530.1D; pAlphaAmyCPOT; ΔSUT019	This study
Q17	CEN.PK 530.1D; pAlphaAmyCPOT; ΔCUT060	This study
Q18	CEN.PK 530.1D; pAlphaAmyCPOT; ΔSUT526	This study
Q19	CEN.PK 530.1D; pAlphaAmyCPOT; ΔSUT067; ΔSUT019	This study

Table S2. Plasmids used in this study.

Plasmid	Relevant characteristics/ genotype	Reference
p416-GPD	CEN6/ARSH4, AmpR, *URA3*, *GPDp*, *CYC1t*	(Mumberg et al., 1995)[2]
p426-GPD	2μm, AmpR, *URA3*, *GPDp*, *CYC1t*	(Mumberg et al., 1995)[2]
pROS13	2μm AmpR *URA3* gRNA-*CAN1*.Y gRNA-*ADE2*.Y, *loxP-KanMX4-loxP*	(Mans et al., 2015)[3]
pAlphaAmyCPOT	CPOTud-(*TPI1*p-*alpha factor leader-amylase gene-TPI1*t)	(Liu et al., 2012)[4]
pGM-PDI1	2 μm, AmpR, *URA3*, *TEF1*p, *ADH1*t, *PGK1*p, *CYC1*t, -(*TEF1*p-*PDI1-ADH1*t)	(Huang et al., 2018)[1]
pCP-AHSA	CPOTud-(*TPI1*p-*alpha factor leader-HSA, human serum albumin gene-TPI1*t)	(Huang et al., 2017)[5]
pCas9	2μm AmpR *TEF1p-Cas9 SNR52p*	(Zhang et al., 2019)[6]
p416-GPD-SFP1	CEN6/ARSH4, AmpR, *URA3, GPDp-SFP1-CYC1t*	This study
p416-PDC1-FaF3H	CEN6/ARSH4, AmpR, *URA3*, *PDC1p-FaF3H-PGK1t*	This study
p416-GPD-tHMG1	CEN6/ARSH4, AmpR, *URA3*, *GPDp- tHMG1-CYC1t*	This study
p416-GPD-SCH9	CEN6/ARSH4, AmpR, *URA3*, *GPDp-SCH9-CYC1t*	This study
p416-GPD-SSB1	CEN6/ARSH4, AmpR, *URA3*, *GPDp-SSB1-CYC1t*	This study
p426-GPD-SRD1	2μm, AmpR, *URA3*, *GPDp-SRD1-CYC1t*	This study
p426-GPD-HMT1	2μm, AmpR, *URA3*, *GPDp-HMT1-CYC1t*	This study
p426-GPD-RRN7	2μm, AmpR, *URA3*, *GPDp-RRN7-CYC1t*	This study
p426-GPD-SPT15	2μm, AmpR, *URA3*, *GPDp-SPT15-CYC1t*	This study
p426-GPD-RRN6	2μm, AmpR, *URA3*, *GPDp-RRN6-CYC1t*	This study
p426-GPD-TGS1	2μm, AmpR, *URA3*, *GPDp-TGS1-CYC1t*	This study
p426-GPD-NOP1	2μm, AmpR, *URA3*, *GPDp-NOP1-CYC1t*	This study
pCas9- ΔCUT461	2μm AmpR *TEF1p-Cas9 SNR52p* gRNA-CUT461	This study

pCas9- ΔSUT019	2μm AmpR *TEF1p-Cas9 SNR52p* gRNA-SUT019	This study
pCas9- ΔSUT311	2μm AmpR *TEF1p-Cas9 SNR52p* gRNA-SUT311	This study
pCas9- ΔCUT462	2μm AmpR *TEF1p-Cas9 SNR52p* gRNA-CUT462	This study
pCas9- ΔSUT493	2μm AmpR *TEF1p-Cas9 SNR52p* gRNA-SUT493	This study
pCas9- ΔncRPR1	2μm AmpR *TEF1p-Cas9 SNR52p* gRNA-ncRPR1	This study
pCas9- ΔCUT378	2μm AmpR *TEF1p-Cas9 SNR52p* gRNA-CUT378	This study
pCas9- ΔSUT842	2μm AmpR *TEF1p-Cas9 SNR52p* gRNA-SUT842	This study
pCas9- ΔSUT126	2μm AmpR *TEF1p-Cas9 SNR52p* gRNA-SUT126	This study
pCas9- ΔSUT496	2μm AmpR *TEF1p-Cas9 SNR52p* gRNA-SUT496	This study
pCas9- ΔCUT672	2μm AmpR *TEF1p-Cas9 SNR52p* gRNA-CUT672	This study
pCas9- ΔCUT084	2μm AmpR *TEF1p-Cas9 SNR52p* gRNA-CUT084	This study
pCas9- ΔSUT602	2μm AmpR *TEF1p-Cas9 SNR52p* gRNA-SUT602	This study
pCas9- ΔSUT035	2μm AmpR *TEF1p-Cas9 SNR52p* gRNA-SUT035	This study
pCas9- ΔSUT509	2μm AmpR *TEF1p-Cas9 SNR52p* gRNA-SUT509	This study
pCas9- ΔSUT591	2μm AmpR *TEF1p-Cas9 SNR52p* gRNA-SUT591	This study
pCas9- ΔSUT361	2μm AmpR *TEF1p-Cas9 SNR52p* gRNA-SUT361	This study
pCas9- ΔSUT218	2μm AmpR *TEF1p-Cas9 SNR52p* gRNA-SUT218	This study
pCas9- ΔCUT060	2μm AmpR *TEF1p-Cas9 SNR52p* gRNA-CUT060	This study
pCas9- ΔSUT526	2μm AmpR *TEF1p-Cas9 SNR52p* gRNA-SUT526	This study
pCas9- ΔTOD6	2μm AmpR *TEF1p-Cas9 SNR52p* gRNA-*TOD6*	This study
pCas9- ΔDOT6	2μm AmpR *TEF1p-Cas9 SNR52p* gRNA-*DOT6*	This study
pCas9- ΔX3	2μm AmpR *TEF1p-Cas9 SNR52p* gRNA-X3	This study
pCas9- ΔRRP6	2μm AmpR *TEF1p-Cas9 SNR52p* gRNA-*RRP6*	This study

pCas9- ΔSEC72	2μm AmpR *TEF1p-Cas9 SNR52p* gRNA-*SEC72*	This study
pCas9- ΔKAR2	2μm AmpR *TEF1p-Cas9 SNR52p* gRNA-*KAR2*	This study
pCas9- ΔPDI1	2μm AmpR *TEF1p-Cas9 SNR52p* gRNA-*PDI1*	This study
pCas9- ΔXI-3	2μm AmpR *TEF1p-Cas9 SNR52p* gRNA-XI-3	This study

References for supporting information

[1] M. Huang, G. Wang, J. Qin, D. Petranovic, J. Nielsen, *Proc Natl Acad Sci U S A* **2018**, 115, E11025.

[2] R. M. Dominik Mumberg, Martin Funk, *Gene* **1995**, 156, 119.

[3] R. Mans, H. M. van Rossum, M. Wijsman, A. Backx, N. G. Kuijpers, M. van den Broek, P. Daran-Lapujade, J. T. Pronk, A. J. van Maris, J. M. Daran, *Fems Yeast Res* **2015**, 15, fov004.

[4] Z. Liu, K. E. Tyo, J. L. Martinez, D. Petranovic, J. Nielsen, *Biotechnol Bioeng* **2012**, 109, 1259.

[5] M. Huang, J. Bao, B. M. Hallström, D. Petranovic, J. Nielsen, *Nat Commun* **2017**, 8, 1131.

[6] Y. Zhang, J. Wang, Z. Wang, Y. Zhang, S. Shi, J. Nielsen, Z. Liu, *Nat Commun* **2019**, 10, 1053.
